# Supplementary material for: Simultaneous inference of phylogenetic and transmission trees in infectious disease outbreaks
Source: PLoS Comput Biol. 2017 May 18;13(5):e1005495. doi: 10.1371/journal.pcbi.1005495 (PMC5436636; doi:10.1371/journal.pcbi.1005495)
Supplement: S1 Results — (DOCX) [file pcbi.1005495.s001.docx]

S1 Results

**Table S1.1. Performance on 25 newly simulated datasets of 50 cases, with shape parameters *a_S_* = *a_G_* = 3.**

|  |  | **Level of prior information on *m_S_*** | | |
| --- | --- | --- | --- | --- |
|  | **Reference ^a^** | **Informative Correct ^b^** | **Uninformative ^c^** | **Informative Wrong ^d^** |
| **MCMC sampling** |  |  |  |  |
| Continuous parameter samples (95% interval of ESS) | | | | |
| *μ* | 3579 ; 6784 | 1176 ; 2181 | 187 ; 1262 | 709 ; 1435 |
| *m_G_* |  | 865 ; 1977 | 528 ; 1468 | 499 ; 1265 |
| *m_S_* |  | 412 ; 822 | 51 ; 149 | 837 ; 1849 |
| *r* |  | 273 ; 450 | 218 ; 411 | 183 ; 345 |
| *t_inf_* | 1008 ; 5432 | 607 ; 4632 | 301 ; 2399 | 319 ; 2683 |
| *phylogenetic tree topology* ^e^ | 1592 ; 3672 | 1422 ; 3000 | 1174 ; 2738 | 900 ; 2339 |
| Infectors (% Fisher’s exact tests accepted) | | | | |
| *between chains* | 99.0% | 98.5% | 98.0% | 99.0% |
| *autocorrelation* | 95.3% | 96.3% | 94.5% | 95.4% |
| **Parameter inference (95% interval of posterior medians)** | | | | |
| log_10_(*μ*) | -4.13 ; -3.92 | -4.11 ; -3.90 | -4.13 ; -3.89 | -4.27 ; -4.03 |
| *m_G_* |  | 0.56 ; 1.10 | 0.56 ; 1.10 | 0.52 ; 1.00 |
| *m_S_* |  | 0.97 ; 1.08 | 0.77 ; 1.43 | 1.89 ; 1.97 |
| *r* |  | 0.66 ; 1.20 | 0.73 ; 1.17 | 0.43 ; 0.90 |
| **Tree inference** |  |  |  |  |
| Infection times (coverage: % of 95% CIs containing the true value) | | | | |
|  | 94.7% | 94.8% | 94.8% | 77.7% |
| Infection time bias (median) | 0.00 yr | -0.03 yr | -0.07 yr | -0.67 yr |
| 95% interval of medians | -0.73 ; 1.25 | -0.93 ; 1.19 | -1.00 ; 1.15 | -2.00 ; 0.52 |
| Infectors (number correct/number identified) | | | | |
| *Edmonds’* | 23.2/50 | 23.2/50 | 23.3/50 | 21.8/50 |
| *MPC* | 22.0/50 | 21.8/50 | 22.0/50 | 19.1/50 |
| *> 50% support* | 14.5/20.7 | 14.0/19.9 | 13.9/19.5 | 7.9/10.2 |
| *> 80% support* | 5.3/6.2 | 4.9/5.8 | 4.9/5.8 | 1.5/1.8 |
| *TransPhylo (Edmonds’)* | 21.3/50 |  |  |  |
| *Outbreaker (Edmonds’)* | 21.2/50 |  |  |  |

Results are based on two MCMC chains of 25,000 samples each; ESS, effective sample size; CI, credible interval; MPC, maximum parent credibility. ^a^ *m_G_*, *m_S_*, *r* = 1; ^b^ *μ_S_* = 1, *σ_S_* = 0.1; ^c^ *μ_S_* = 1, *σ_S_* = ∞; ^d^ *μ_S_* = 2, *σ_S_* = 0.1

**Table S1.2. Tree inference with incomplete data, with 25 newly simulated datasets of 50 cases, of which 40 observed, simulated with shape parameters *a_S_* = *a_G_* = 3.**

|  | **Complete data** | **Incomplete data** | |
| --- | --- | --- | --- |
|  | **Reference ^a^** | **Reference ^a^** | **Uninformative ^b^** |
| **Tree inference** |  |  |  |
| Infection times (coverage: % of 95% CIs containing the true value) | | | |
|  | 94.7% | 81.7% | 80.1% |
| Infection time bias (median) | | | |
| - all cases | 0.00 yr (-0.73 ; 1.25) | -0.13 yr (-2.02 ; 1.15) | -0.27 yr (-2.30 ; 1.00) |
| - infectors in data ^c^ |  | -0.12 yr (-1.98 ; 1.09) | -0.25 yr (-2.21 ; 0.97) |
| - orphans ^d^ |  | -0.23 yr (-2.16 ; 1.32) | -0.33 yr (-2.53 ; 1.06) |
|  |  |  |  |
| Infectors (number correct/number identified) | | | |
| Edmonds’ |  |  |  |
| - all cases | 23.2/50 (46%) | 16.0/40 (40%) | 15.8/40 (40%) |
| - infectors in data ^c^ |  | 16.0/31.0 (51%) | 15.8/31.0 (51%) |
| - orphans (ancestors)^d^ |  | 3.0/9.0 (33%) | 3.0/9.0 (33%) |
| *>50% support* |  |  |  |
| - all cases | 14.5/20.7 (70%) | 11.2/18.9 (60%) | 10.3/16.7 (61%) |
| - infectors in data ^c^ |  | 11.2/15.4 (73%) | 10.3/13.6 (75%) |
| - orphans (ancestors)^d^ |  | 1.6/3.4 (47%) | 1.4/3.1 (47%) |
| *>80% support* |  |  |  |
| - all cases | 5.3/6.2 (85%) | 4.4/5.9 (75%) | 3.6/4.8 (75%) |
| - infectors in data ^c^ |  | 4.4/4.8 (91%) | 3.6/4.0 (90%) |
| - orphans (ancestors)^d^ |  | 0.6/1.0 (58%) | 0.5/0.8 (60%) |

Results are based on two MCMC chains of 25,000 samples each; CI, credible interval. ^a^ *m_G_*, *m_S_*, *r* = 1; ^b^ *μ_S_* = 1, *σ_S_* = ∞; ^c^ only cases whose infector was in the dataset; ^d^ only orphans (infector not in the dataset), counting identified ancestors of the true infector.

**Table S1.3. Performance on 25 newly simulated datasets of 20 cases, with shape parameters *a_S_* = *a_G_* = 10.**

|  |  | **Level of prior information on *m_S_*** | | |
| --- | --- | --- | --- | --- |
|  | **Reference ^a^** | **Informative Correct ^b^** | **Uninformative ^c^** | **Informative Wrong ^d^** |
| **MCMC sampling** |  |  |  |  |
| Continuous parameter samples (95% interval of ESS) | | | | |
| *μ* | 7691 ; 10204 | 2576 ; 5118 | 162 ; 2596 | 2340 ; 5366 |
| *m_G_* |  | 1563 ; 5832 | 801 ; 3543 | 1231 ; 4410 |
| *m_S_* |  | 844 ; 2234 | 29 ; 221 | 1003 ; 3086 |
| *r* |  | 689 ; 1146 | 616 ; 1150 | 590 ; 992 |
| *t_inf_* | 2229 ; 12824 | 1457 ; 7865 | 107 ; 682 | 686 ; 5419 |
| *phylogenetic tree topology* ^e^ | 2106 ; 5478 | 1974 ; 4892 | 1822 ; 4458 | 1750 ; 3823 |
| Infectors (% Fisher’s exact tests accepted) | | | | |
| *between chains* | 97.2% | 96.0% | 95.4% | 96.4% |
| *autocorrelation* | 95.8% | 95.0% | 92.4% | 97.6% |
| **Parameter inference (95% interval of posterior medians)** | | | | |
| log_10_(*μ*) | -4.22 ; -3.89 | -4.22 ; -3.86 | -4.33 ; -3.82 | -4.41 ; -4.06 |
| *m_G_* |  | 0.60 ; 1.20 | 0.60 ; 1.22 | 0.54 ; 1.11 |
| *m_S_* |  | 0.95 ; 1.04 | 0.56 ; 2.00 | 1.95 ; 2.01 |
| *r* |  | 0.61 ; 1.21 | 0.67 ; 1.13 | 0.53 ; 0.98 |
| **Tree inference** |  |  |  |  |
| Infection times (coverage: % of 95% CIs containing the true value) | | | | |
|  | 94.0% | 94.0% | 95.8% | 41.6% |
| Infection time bias (median) | 0.00 yr | -0.02 yr | -0.02 yr | -0.91 yr |
| 95% interval of medians | -0.53 ; 0.67 | -0.56 ; 0.61 | -1.21 ; 0.81 | -1.69 ; -0.27 |
| Infectors (number correct/number identified) | | | | |
| *Edmonds’* | 13.3/20 | 13.3/20 | 13.1/20 | 13.6/20 |
| *MPC* | 13.5/20 | 13.3/20 | 13.2/20 | 12.9/20 |
| *> 50% support* | 11.0/13.4 | 10.8/13.4 | 10.1/12.6 | 7.8/8.7 |
| *> 80% support* | 6.4/6.5 | 6.2/6.3 | 5.8/5.9 | 3.1/3.1 |
| *TransPhylo (Edmonds’)* | 12.8/20 |  |  |  |
| *Outbreaker (Edmonds’)* | 11.5/20 |  |  |  |

Results are based on two MCMC chains of 25,000 samples each; ESS, effective sample size; CI, credible interval; MPC, maximum parent credibility. ^a^ *m_G_*, *m_S_*, *r* = 1; ^b^ *μ_S_* = 1, *σ_S_* = 0.1; ^c^ *μ_S_* = 1, *σ_S_* = ∞; ^d^ *μ_S_* = 2, *σ_S_* = 0.1

**Table S1.4. Tree inference with incomplete data, with 25 newly simulated datasets of 20 cases, of which 16 observed, simulated with shape parameters *a_S_* = *a_G_* = 10.**

|  | **Complete data** | **Incomplete data** | |
| --- | --- | --- | --- |
|  | **Reference ^a^** | **Reference ^a^** | **Uninformative ^b^** |
| **Tree inference** |  |  |  |
| Infection times (coverage: % of 95% CIs containing the true value) | | | |
|  | 94.7% | 83.3% | 82.5% |
| Infection time bias (median) | | | |
| - all cases | 0.00 yr (-0.53 ; 0.67) | -0.07 yr (-1.73 ; 0.66) | -0.20 yr (-2.60 ; 0.77) |
| - infectors in data ^c^ |  | -0.07 yr (-1.78 ; 0.58) | -0.22 yr (-2.59 ; 0.72) |
| - orphans ^d^ |  | -0.10 yr (-1.48 ; 0.67) | -0.16 yr (-2.51 ; 0.77) |
|  |  |  |  |
| Infectors (number correct/number identified) | | | |
| Edmonds’ |  |  |  |
| - all cases | 13.3/20 (67%) | 9.1/16 (57%) | 8.9/16 (56%) |
| - infectors in data ^c^ |  | 9.1/13.0 (70%) | 8.9/13.0 (69%) |
| - orphans (ancestors)^d^ |  | 1.1/3.0 (36%) | 1.2/3.0 (39%) |
| *>50% support* |  |  |  |
| - all cases | 11.0/13.4 (82%) | 7.8/11.9 (66%) | 6.9/10.4 (66%) |
| - infectors in data ^c^ |  | 7.8/9.6 (81%) | 6.9/8.5 (81%) |
| - orphans (ancestors)^d^ |  | 1.0/2.2 (45%) | 1.0/1.9 (51%) |
| *>80% support* |  |  |  |
| - all cases | 6.4/6.5 (99%) | 5.1/6.0 (85%) | 4.0/4.9 (81%) |
| - infectors in data ^c^ |  | 5.1/5.3 (97%) | 4.0/4.1 (96%) |
| - orphans (ancestors)^d^ |  | 0.5/0.7 (72%) | 0.5/0.8 (68%) |

Results are based on two MCMC chains of 25,000 samples each; CI, credible interval. ^a^ *m_G_*, *m_S_*, *r* = 1; ^b^ *μ_S_* = 1, *σ_S_* = ∞; ^c^ only cases whose infector was in the dataset; ^d^ only orphans (infector not in the dataset), counting identified ancestors of the true infector.

**Table S1.5. Performance on 25 newly simulated datasets of 20 cases, with shape parameters *a_S_* = *a_G_* = 3.**

|  |  | **Level of prior information on *m_S_*** | | |
| --- | --- | --- | --- | --- |
|  | **Reference ^a^** | **Informative Correct ^b^** | **Uninformative ^c^** | **Informative Wrong ^d^** |
| **MCMC sampling** |  |  |  |  |
| Continuous parameter samples (95% interval of ESS) | | | | |
| *μ* | 5302 ; 8611 | 2417 ; 4942 | 268 ; 3459 | 1287 ; 3000 |
| *m_G_* |  | 807 ; 5194 | 392 ; 4874 | 559 ; 3058 |
| *m_S_* |  | 1118 ; 4341 | 69 ; 374 | 2912 ; 7231 |
| *r* |  | 694 ; 1177 | 694 ; 1140 | 551 ; 982 |
| *t_inf_* | 1232 ; 7312 | 688 ; 6326 | 164 ; 2142 | 298 ; 3935 |
| *phylogenetic tree topology* ^e^ | 2173 ; 4593 | 1965 ; 3809 | 1403 ; 3717 | 1284 ; 3000 |
| Infectors (% Fisher’s exact tests accepted) | | | | |
| *between chains* | 95.8% | 95.2% | 97.0% | 96.8% |
| *autocorrelation* | 96.6% | 95.8% | 95.8% | 96.0% |
| **Parameter inference (95% interval of posterior medians)** | | | | |
| log_10_(*μ*) | -4.15 ; -3.88 | -4.14 ; -3.87 | -4.29 ; -3.80 | -4.32 ; -4.02 |
| *m_G_* |  | 0.52 ; 1.18 | 0.47 ; 1.27 | 0.42 ; 1.13 |
| *m_S_* |  | 0.95 ; 1.06 | 0.42 ; 1.83 | 1.93 ; 2.00 |
| *r* |  | 0.71 ; 1.11 | 0.78 ; 1.10 | 0.63 ; 0.93 |
| **Tree inference** |  |  |  |  |
| Infection times (coverage: % of 95% CIs containing the true value) | | | | |
|  | 95.2% | 94.0% | 93.4% | 80.4% |
| Infection time bias (median) | 0.01 yr | 0.01 yr | 0.05 yr | -0.64 yr |
| 95% interval of medians | -0.77 ; 1.11 | -0.90 ; 1.11 | -1.30 ; 1.22 | -2.19 ; 0.46 |
| Infectors (number correct/number identified) | | | | |
| *Edmonds’* | 10.7/20 | 10.5/20 | 10.2/20 | 10.2/20 |
| *MPC* | 10.2/20 | 10.5/20 | 10.0/20 | 9.6/20 |
| *> 50% support* | 6.6/8.8 | 6.4/8.6 | 6.4/8.3 | 4.2/5.1 |
| *> 80% support* | 2.8/3.2 | 2.7/3.0 | 2.9/3.2 | 0.8/0.9 |
| *TransPhylo (Edmonds’)* | 8.6/20 |  |  |  |
| *Outbreaker (Edmonds’)* | 9.2/20 |  |  |  |

Results are based on two MCMC chains of 25,000 samples each; ESS, effective sample size; CI, credible interval; MPC, maximum parent credibility. ^a^ *m_G_*, *m_S_*, *r* = 1; ^b^ *μ_S_* = 1, *σ_S_* = 0.1; ^c^ *μ_S_* = 1, *σ_S_* = ∞; ^d^ *μ_S_* = 2, *σ_S_* = 0.1

**Table S1.6. Tree inference with incomplete data, with 25 newly simulated datasets of 20 cases, of which 16 observed, simulated with shape parameters *a_S_* = *a_G_* = 3.**

|  | **Complete data** | **Incomplete data** | |
| --- | --- | --- | --- |
|  | **Reference ^a^** | **Reference ^a^** | **Uninformative ^b^** |
| **Tree inference** |  |  |  |
| Infection times (coverage: % of 95% CIs containing the true value) | | | |
|  | 95.2% | 82.8% | 83.5% |
| Infection time bias (median) | | | |
| - all cases | 0.01 yr (-0.77 ; 1.11) | -0.07 yr (-1.51 ; 1.15) | -0.10 yr (-1.69 ; 1.24) |
| - infectors in data ^c^ |  | -0.04 yr (-1.42 ; 1.25) | -0.05 yr (-1.65 ; 1.22) |
| - orphans ^d^ |  | -0.14 yr (-1.53 ; 0.94) | -0.21 yr (-1.90 ; 1.19) |
|  |  |  |  |
| Infectors (number correct/number identified) | | | |
| Edmonds’ |  |  |  |
| - all cases | 10.7/20 (53%) | 7.2/16 (45%) | 7.1/16 (44%) |
| - infectors in data ^c^ |  | 7.2/11.9 (60%) | 7.1/11.9 (60%) |
| - orphans (ancestors)^d^ |  | 1.3/4.1 (32%) | 1.2/4.1 (30%) |
| *>50% support* |  |  |  |
| - all cases | 6.6/8.8 (74%) | 5.3/8.2 (65%) | 5.0/8.1 (62%) |
| - infectors in data ^c^ |  | 5.3/6.7 (79%) | 5.0/6.6 (76%) |
| - orphans (ancestors)^d^ |  | 0.6/1.4 (44%) | 0.6/1.5 (43%) |
| *>80% support* |  |  |  |
| - all cases | 2.8/3.2 (89%) | 2.4/3.0 (78%) | 2.2/2.8 (80%) |
| - infectors in data ^c^ |  | 2.4/2.6 (91%) | 2.2/2.4 (92%) |
| - orphans (ancestors)^d^ |  | 0.2/0.4 (45%) | 0.2/0.4 (44%) |

Results are based on two MCMC chains of 25,000 samples each; CI, credible interval. ^a^ *m_G_*, *m_S_*, *r* = 1; ^b^ *μ_S_* = 1, *σ_S_* = ∞; ^c^ only cases whose infector was in the dataset; ^d^ only orphans (infector not in the dataset), counting identified ancestors of the true infector.

**Table S1.7. Performance on 25 published simulated datasets in populations of size 50.**

|  | ***Slow Clock* simulations** | | ***Fast Clock* simulations** | |
| --- | --- | --- | --- | --- |
| **Prior information** | **Uninformative ^a^** | **Informative ^b^** | **Uninformative ^a^** | **Informative ^b^** |
| **MCMC sampling** |  |  |  |  |
| Continuous parameter samples (95% interval of ESS) | | | | |
| *μ* | 40 ; 314 | 401 ; 951 | 93 ; 726 | 90 ; 916 |
| *m_G_* | 1375 ; 4310 | 1948 ; 4531 | 3316 ; 11526 | 2999 ; 12748 |
| *m_S_* | 5 ; 31 | 43 ; 87 | 111 ; 364 | 170 ; 461 |
| *r* | 189 ; 335 | 220 ; 369 | 158 ; 534 | 184 ; 613 |
| *t_inf_* | 25 ; 189 | 222 ; 559 | 268 ; 2443 | 295 ; 2796 |
| *phylogenetic tree topology* ^c^ |  | 1501 ; 4331 | 87 ; 689 | 83 ; 796 |
| Infectors (% Fisher’s exact tests accepted) | | | | |
| *between chains* | 94.9% | 97.4% | 97.4% | 97.6% |
| *autocorrelation* | 94.4% | 96.4% | 94.9% | 96.0% |
| **Parameter inference** (95% interval of posterior medians) | | | | |
| log_10_(*μ*) | -5.18 ; -4.74 | -4.95 ; -4.78 | -3.19 ; -3.15 | -3.20 ; -3.16 |
| *m_G_* | 3.6 ; 5.8 | 3.7 ; 5.7 | 4.72 ; 6.13 | 4.7 ; 6.1 |
| *m_S_* | 8.8 ; 24.0 | 11.2 ; 12.6 | 11.20 ; 12.63 | 11.4 ; 12.6 |
| *r* | 0.43 ; 0.85 | 0.36 ; 0.77 | 0.29 ; 1.23 | 0.30 ; 1.2 |
| **Tree inference** |  |  |  |  |
| Infection times (coverage: % of 95% CIs containing the true value) | | | | |
|  | 87.8% | 97.8% | 92.9% | 94.2% |
| Infection time bias (median) | 0.33 days | 0.10 days | 0.14 days | 0.01 days |
| >95% interval of medians | (-13.95 ; 4.20) | (-1.98 ; 2.28) | (-1.99 ; 2.19) | (-2.00 ; 1.97) |
| Infectors (number correct/number identified) | | | | |
| *Edmonds’* | 30.8/49.3 | 30.8/49.3 | 45.4/49.3 | 45.5/49.3 |
| *MPC* | 30.6/49.3 | 29.9/49.3 | 45.4/49.3 | 45.5/49.3 |
| *>50% support* | 24.8/30.4 | 25.2/30.9 | 45.2/48.9 | 45.4/48.7 |
| *>80% support* | 17.9/19.1 | 18.5/19.7 | 41.0/42.3 | 40.9/42.1 |

Results are based on two MCMC chains of 25,000 samples each. The mean outbreak size was 49.3 cases; ESS, effective sample size; CI, credible interval; MPC, maximum parent credibility. ^a^ *a_S_* = 144, *μ_S_* = 1, *σ_S_* = ∞; ^b^ *a_S_* = 144, *μ_S_* = 12, *σ_S_* = 1

**Table S1.8. Edmonds’ consensus tree for the Mtb data.**

infector support inf.times.Q2.5 inf.times.Q50 inf.times.Q97.5

K01 K29 0.15626 2007-04-08 2008-02-22 2009-04-10

K02 index 0.33340 2006-12-29 2007-09-09 2008-03-02

K03 K34 0.14812 2008-06-02 2009-07-02 2010-04-16

K04 K22 0.25916 2007-07-26 2008-04-23 2008-12-13

K05 K19 0.52924 2007-04-30 2008-02-20 2008-10-17

K06 K20 0.35862 2008-02-28 2008-12-01 2009-06-26

K10 K22 0.25740 2007-08-08 2008-04-23 2008-12-14

K11 K06 0.25110 2008-04-22 2009-04-02 2010-05-04

K14 K34 0.14706 2008-05-26 2009-06-29 2010-04-07

K15 K34 0.19346 2008-05-09 2009-04-26 2010-01-12

K16 K29 0.19792 2007-02-21 2007-12-19 2008-09-09

K17 K01 0.18266 2007-04-24 2008-04-16 2009-09-14

K18 K06 0.25132 2008-04-13 2009-03-22 2010-05-08

K19 K02 0.10642 2007-05-06 2008-02-05 2008-09-18

K20 K30 0.49762 2008-01-27 2008-09-22 2009-02-06

K21 K22 0.23226 2007-08-26 2008-05-30 2009-05-26

K22 K29 0.06496 2007-07-21 2008-03-26 2008-09-15

K24 K03 0.11520 2008-06-07 2009-09-13 2010-07-29

K25 K31 0.11352 2008-06-06 2009-09-23 2010-08-15

K27 K22 0.22656 2007-07-04 2008-04-29 2009-04-23

K28 K17 0.19958 2007-06-03 2008-05-29 2009-12-21

K29 K02 0.24830 2007-02-11 2007-11-02 2008-04-28

K30 K22 0.16816 2007-12-18 2008-07-12 2008-11-11

K31 K34 0.17322 2008-05-04 2009-06-12 2010-03-05

K32 K28 0.21352 2007-05-10 2008-06-06 2010-04-15

K33 K02 0.21188 2007-02-04 2007-11-17 2008-07-09

K34 K35 0.30340 2008-04-30 2009-01-30 2009-09-27

K35 K02 0.21892 2007-06-25 2008-01-19 2008-05-10

K36 K25 0.10734 2008-06-10 2009-10-23 2010-10-15

K37 K14 0.10870 2008-05-27 2009-08-30 2010-10-01

K38 K03 0.10686 2008-06-10 2009-09-12 2010-10-20

K39 K11 0.28218 2008-04-21 2009-04-20 2010-10-09

K40 K31 0.10624 2008-06-14 2009-09-21 2010-10-30

**Table S1.9. Edmonds’ consensus tree for the MRSA data.**

infector support inf.times.Q2.5 inf.times.Q50 inf.times.Q97.5

P22 M_P22 0.64052 2010-04-08 2010-05-31 2010-06-24

HCW_A P5 0.15452 2009-12-23 2010-02-05 2010-05-10

HCW_B P16 0.51530 2010-02-25 2010-04-20 2010-07-11

M_P22 P15 0.29978 2010-04-07 2010-05-19 2010-06-08

M_P7 P11 0.71872 2010-01-21 2010-02-24 2010-03-02

P30 P25 0.22264 2010-05-22 2010-07-18 2010-08-16

P32 P31 0.35616 2010-05-10 2010-07-07 2010-08-13

P25 P26 0.20632 2010-05-19 2010-07-18 2010-08-14

P31 P20 0.32072 2010-05-07 2010-07-06 2010-08-06

P29 P19 0.35392 2010-04-15 2010-06-13 2010-07-15

P26 P23 0.22016 2010-05-12 2010-06-27 2010-07-11

P24 P23 0.21676 2010-05-03 2010-06-19 2010-07-04

P27 P15 0.12418 2010-04-22 2010-06-07 2010-07-01

P28 P27 0.22096 2010-05-01 2010-06-17 2010-07-01

P23 P27 0.22834 2010-05-02 2010-06-15 2010-06-29

P15 M_P7 0.42072 2010-03-08 2010-04-13 2010-06-02

P20 P19 0.53374 2010-04-29 2010-06-09 2010-06-20

P19 P16 0.35266 2010-04-19 2010-05-25 2010-06-06

P17 P18 0.59914 2010-04-06 2010-05-21 2010-06-06

P18 P15 0.29770 2010-04-04 2010-05-13 2010-05-30

P16 P7 0.13306 2010-02-26 2010-04-15 2010-05-02

P9 P14 0.23798 2009-12-12 2010-02-11 2010-04-16

P14 P5 0.11550 2009-12-12 2010-02-10 2010-04-02

P11 HCW_A 0.26394 2010-01-12 2010-02-09 2010-02-17

P12 P5 0.17432 2009-12-18 2010-01-27 2010-02-13

P10 P8 0.45520 2009-12-31 2010-02-26 2010-03-27

P8 P12 0.38560 2010-01-02 2010-02-12 2010-02-27

P3 index 0.24910 2009-12-01 2010-01-08 2010-01-27

P1 P3 0.17498 2009-12-12 2010-01-25 2010-02-08

P13 P3 0.16780 2009-12-15 2010-02-04 2010-02-21

P2 P3 0.17946 2009-12-12 2010-01-28 2010-02-14

P21 P15 0.17554 2010-02-23 2010-05-13 2010-06-12

P4 P3 0.16200 2009-12-15 2010-02-04 2010-02-21

P5 P3 0.22240 2009-12-06 2010-01-11 2010-01-23

P6 P3 0.15380 2009-12-17 2010-02-05 2010-02-22

P7 P3 0.16828 2009-12-18 2010-02-10 2010-02-27

**Table S1.10. Edmonds’ consensus tree for the FMD2001 data.**

infector support inf.times.Q2.5 inf.times.Q50 inf.times.Q97.5

A index 0.58528 2001-02-18 2001-03-09 2001-03-27

K B 0.67390 2001-03-11 2001-03-23 2001-03-29

L K 0.76122 2001-03-19 2001-03-31 2001-04-06

N A 0.93724 2001-03-02 2001-03-31 2001-04-08

O B 0.55948 2001-03-14 2001-03-28 2001-04-08

B A 0.46862 2001-02-23 2001-03-14 2001-03-27

F K 0.90876 2001-03-26 2001-04-08 2001-04-17

E L 0.86310 2001-03-26 2001-04-10 2001-04-19

C O 0.51908 2001-03-19 2001-04-03 2001-04-14

P C 0.42964 2001-03-21 2001-04-09 2001-04-25

M D 0.79634 2001-04-14 2001-05-06 2001-05-14

G F 0.82408 2001-04-14 2001-05-03 2001-05-11

D C 0.32780 2001-04-08 2001-04-27 2001-05-08

I G 0.86006 2001-05-10 2001-05-18 2001-05-25

J I 0.82894 2001-05-13 2001-05-26 2001-05-31

**Table S1.11. Edmonds’ consensus tree for the FMD2007 data.**

infector support inf.times.Q2.5 inf.times.Q50 inf.times.Q97.5

IP1b/1 IP1b/2 0.37692 2007-07-18 2007-07-27 2007-08-01

IP1b/2 index 0.37814 2007-07-16 2007-07-27 2007-08-02

IP2b IP1b/1 0.49014 2007-07-24 2007-08-01 2007-08-04

IP2c IP1b/1 0.42344 2007-07-16 2007-07-29 2007-08-04

IP3b IP4b 0.47404 2007-08-28 2007-09-06 2007-09-10

IP3c IP4b 0.54972 2007-08-20 2007-09-04 2007-09-12

IP4b IP5 0.48308 2007-08-20 2007-09-02 2007-09-10

IP5 IP2b 0.91826 2007-08-06 2007-08-18 2007-09-07

IP6b IP3b 0.81508 2007-08-31 2007-09-12 2007-09-18

IP7 IP3b 0.56662 2007-09-07 2007-09-15 2007-09-21

IP8 IP7 0.66332 2007-09-07 2007-09-19 2007-09-26

**Table S1.11. Edmonds’ consensus tree for the H7N7 data (all genes).**

infector support inf.times.Q2.5 inf.times.Q50 inf.times.Q97.5

EPI_ISL_82373 EPI_ISL_82375 0.144876 2003-02-14 2003-02-20 2003-02-24

EPI_ISL_82374 EPI_ISL_82373 0.250016 2003-02-15 2003-02-21 2003-02-24

EPI_ISL_83984 EPI_ISL_82381 0.220380 2003-02-16 2003-02-21 2003-02-24

EPI_ISL_83985 EPI_ISL_83984 0.247824 2003-02-17 2003-02-21 2003-02-24

EPI_ISL_67934 EPI_ISL_82373 0.249432 2003-02-15 2003-02-21 2003-02-24

EPI_ISL_83986 EPI_ISL_82375 0.135300 2003-02-14 2003-02-20 2003-02-25

EPI_UNK_7 EPI_ISL_83985 0.131072 2003-02-16 2003-02-23 2003-02-27

EPI_ISL_82375 index 0.481616 2003-02-11 2003-02-15 2003-02-21

EPI_ISL_83988 EPI_ISL_82381 0.492792 2003-02-13 2003-02-19 2003-02-24

EPI_ISL_82376 EPI_ISL_82373 0.136352 2003-02-18 2003-02-23 2003-02-27

EPI_ISL_83989 EPI_ISL_83988 0.658912 2003-02-20 2003-02-24 2003-02-26

EPI_ISL_68268 EPI_ISL_67934 0.206508 2003-02-18 2003-02-23 2003-02-26

EPI_ISL_83990 EPI_ISL_83986 0.337344 2003-02-17 2003-02-23 2003-02-26

EPI_ISL_68269 EPI_ISL_82376 0.496628 2003-02-17 2003-02-23 2003-02-27

EPI_ISL_82471 EPI_ISL_83984 0.147248 2003-02-18 2003-02-23 2003-02-26

EPI_ISL_82377 EPI_ISL_82380 0.472828 2003-02-20 2003-02-25 2003-03-01

EPI_ISL_82378 EPI_ISL_82406 0.687848 2003-02-19 2003-02-24 2003-02-27

EPI_ISL_82379 EPI_ISL_83985 0.686968 2003-02-23 2003-02-26 2003-02-28

EPI_ISL_82380 EPI_ISL_82471 0.235364 2003-02-20 2003-02-25 2003-03-01

EPI_ISL_82381 EPI_ISL_82375 0.284452 2003-02-12 2003-02-17 2003-02-25

EPI_ISL_82382 EPI_ISL_82405 0.166148 2003-02-20 2003-02-25 2003-03-01

EPI_ISL_82383 EPI_ISL_82373 0.130364 2003-02-19 2003-02-25 2003-03-01

EPI_ISL_82384 EPI_ISL_83986 0.168240 2003-02-20 2003-02-25 2003-03-01

EPI_ISL_82385 EPI_ISL_82384 0.427180 2003-02-20 2003-02-25 2003-03-01

EPI_ISL_68270 EPI_ISL_83986 0.278056 2003-02-20 2003-02-26 2003-03-02

EPI_ISL_68279 EPI_ISL_83984 0.764920 2003-02-19 2003-02-24 2003-02-28

EPI_ISL_82386 EPI_ISL_83986 0.355908 2003-02-17 2003-02-24 2003-02-28

EPI_ISL_82387 EPI_ISL_83986 0.338132 2003-02-15 2003-02-22 2003-02-27

EPI_ISL_82388 EPI_ISL_82471 0.448888 2003-02-19 2003-02-25 2003-03-01

EPI_ISL_68280 EPI_ISL_83989 0.476588 2003-02-21 2003-02-26 2003-03-02

EPI_ISL_82389 EPI_ISL_68268 0.151744 2003-02-21 2003-02-26 2003-03-02

EPI_ISL_82390 EPI_ISL_83990 0.966472 2003-02-23 2003-02-28 2003-03-03

EPI_ISL_82391 EPI_ISL_83985 0.302904 2003-02-24 2003-02-28 2003-03-03

EPI_ISL_83991 EPI_ISL_83984 0.571848 2003-02-22 2003-02-26 2003-03-02

EPI_ISL_68281 EPI_ISL_82383 0.621416 2003-02-20 2003-02-26 2003-03-02

EPI_ISL_82392 EPI_ISL_83985 0.448016 2003-02-23 2003-02-27 2003-03-02

EPI_ISL_82393 EPI_ISL_82471 0.427872 2003-02-20 2003-02-26 2003-03-02

EPI_ISL_68289 EPI_ISL_83986 0.174604 2003-02-25 2003-03-02 2003-03-05

EPI_ISL_68300 EPI_ISL_68303 0.250108 2003-02-26 2003-03-03 2003-03-06

EPI_ISL_68282 EPI_ISL_83985 0.582760 2003-02-20 2003-02-25 2003-03-03

EPI_ISL_82394 EPI_ISL_67934 0.160876 2003-02-20 2003-02-25 2003-03-01

EPI_ISL_82395 EPI_ISL_68270 0.867144 2003-02-24 2003-03-02 2003-03-05

EPI_ISL_82396 EPI_ISL_82382 0.161592 2003-02-24 2003-03-01 2003-03-05

EPI_ISL_82397 EPI_ISL_82379 0.984364 2003-03-01 2003-03-04 2003-03-07

EPI_ISL_68306 EPI_ISL_68268 0.119844 2003-02-25 2003-03-02 2003-03-05

EPI_ISL_82398 EPI_ISL_82406 0.947988 2003-02-24 2003-02-27 2003-03-02

EPI_ISL_83992 EPI_ISL_83989 0.448176 2003-02-21 2003-02-26 2003-03-02

EPI_ISL_68308 EPI_ISL_82382 0.137444 2003-02-23 2003-03-01 2003-03-05

EPI_ISL_68310 EPI_ISL_83991 0.693256 2003-02-27 2003-03-04 2003-03-07

EPI_ISL_82399 EPI_ISL_83992 0.493032 2003-02-24 2003-03-02 2003-03-06

EPI_ISL_82400 EPI_ISL_82398 0.778284 2003-02-26 2003-03-03 2003-03-07

EPI_ISL_82401 EPI_ISL_82380 0.430348 2003-02-27 2003-03-03 2003-03-07

EPI_UNK_53 EPI_ISL_68289 0.055056 2003-02-25 2003-03-03 2003-03-08

EPI_ISL_82402 EPI_ISL_68289 0.654548 2003-03-03 2003-03-06 2003-03-09

EPI_ISL_68271 EPI_ISL_83984 0.408700 2003-02-23 2003-03-01 2003-03-07

EPI_ISL_68272 EPI_ISL_84003 0.641540 2003-02-28 2003-03-06 2003-03-09

EPI_ISL_68283 EPI_ISL_68282 0.515072 2003-02-22 2003-02-28 2003-03-05

EPI_ISL_82403 EPI_ISL_83991 0.601528 2003-02-28 2003-03-04 2003-03-07

EPI_ISL_82404 EPI_ISL_82428 0.898064 2003-02-26 2003-03-04 2003-03-08

EPI_ISL_82405 EPI_ISL_82373 0.229600 2003-02-16 2003-02-22 2003-02-25

EPI_ISL_82406 EPI_ISL_83984 0.214460 2003-02-18 2003-02-22 2003-02-25

EPI_ISL_82407 EPI_ISL_82391 0.855084 2003-02-27 2003-03-04 2003-03-07

EPI_ISL_68301 EPI_ISL_68300 0.426804 2003-03-02 2003-03-07 2003-03-11

EPI_ISL_82408 EPI_ISL_68289 0.365004 2003-03-04 2003-03-08 2003-03-11

EPI_ISL_68284 EPI_ISL_68309 0.480832 2003-02-25 2003-03-04 2003-03-09

EPI_ISL_68309 EPI_ISL_82378 0.638816 2003-02-23 2003-02-28 2003-03-05

EPI_ISL_68273 EPI_ISL_82396 0.170340 2003-02-25 2003-03-03 2003-03-07

EPI_ISL_68313 EPI_ISL_82392 0.459504 2003-02-27 2003-03-05 2003-03-08

EPI_ISL_68330 EPI_ISL_68300 0.283440 2003-03-05 2003-03-08 2003-03-11

EPI_ISL_68302 EPI_ISL_68300 0.367324 2003-03-03 2003-03-08 2003-03-11

EPI_ISL_68311 EPI_ISL_82403 0.397708 2003-03-03 2003-03-08 2003-03-11

EPI_ISL_83993 EPI_ISL_82396 0.222712 2003-02-27 2003-03-05 2003-03-08

EPI_ISL_83994 EPI_ISL_83989 0.961380 2003-02-28 2003-03-04 2003-03-10

EPI_ISL_68285 EPI_ISL_68272 0.494828 2003-03-03 2003-03-08 2003-03-12

EPI_ISL_83995 EPI_ISL_68300 0.250368 2003-03-04 2003-03-09 2003-03-12

EPI_ISL_68274 EPI_ISL_82375 0.857512 2003-02-16 2003-02-25 2003-03-04

EPI_UNK_77 EPI_ISL_82386 0.064848 2003-02-23 2003-02-28 2003-03-04

EPI_ISL_82409 EPI_ISL_83985 0.623096 2003-02-23 2003-02-26 2003-03-01

EPI_ISL_83996 EPI_ISL_82384 0.426692 2003-02-24 2003-03-01 2003-03-05

EPI_ISL_82410 EPI_ISL_82384 0.364568 2003-02-26 2003-03-03 2003-03-07

EPI_ISL_83997 EPI_ISL_82402 0.317720 2003-03-05 2003-03-09 2003-03-12

EPI_ISL_82411 EPI_ISL_82392 0.361828 2003-02-25 2003-03-01 2003-03-05

EPI_ISL_83998 EPI_ISL_82409 0.532568 2003-03-02 2003-03-07 2003-03-11

EPI_ISL_68303 EPI_ISL_83984 0.203400 2003-02-24 2003-03-03 2003-03-10

EPI_ISL_83999 EPI_ISL_82411 0.964160 2003-03-02 2003-03-07 2003-03-10

EPI_ISL_84000 EPI_ISL_68313 0.420648 2003-03-05 2003-03-10 2003-03-14

EPI_ISL_82412 EPI_ISL_82403 0.329680 2003-03-04 2003-03-09 2003-03-13

EPI_UNK_88 EPI_ISL_84011 0.101580 2003-03-03 2003-03-10 2003-03-13

EPI_ISL_82413 EPI_ISL_82401 0.327204 2003-02-27 2003-03-04 2003-03-09

EPI_ISL_68299 EPI_ISL_82402 0.299236 2003-03-06 2003-03-09 2003-03-13

EPI_ISL_82414 EPI_ISL_83993 0.297488 2003-03-03 2003-03-09 2003-03-13

EPI_ISL_82415 EPI_ISL_68300 0.262792 2003-03-04 2003-03-08 2003-03-12

EPI_ISL_84001 EPI_ISL_84006 0.748688 2003-03-04 2003-03-09 2003-03-13

EPI_ISL_68332 EPI_ISL_68306 0.613812 2003-03-04 2003-03-08 2003-03-10

EPI_ISL_82416 EPI_ISL_82399 0.383796 2003-02-28 2003-03-06 2003-03-11

EPI_ISL_82417 EPI_ISL_68306 0.669340 2003-03-03 2003-03-07 2003-03-11

EPI_ISL_84002 EPI_ISL_68300 0.432280 2003-03-02 2003-03-07 2003-03-11

EPI_ISL_84003 EPI_ISL_82385 0.280404 2003-02-27 2003-03-03 2003-03-07

EPI_ISL_82418 EPI_ISL_82386 0.273212 2003-03-04 2003-03-10 2003-03-14

EPI_ISL_82419 EPI_ISL_83993 0.257828 2003-03-04 2003-03-10 2003-03-15

EPI_ISL_84004 EPI_ISL_82403 0.293412 2003-03-04 2003-03-10 2003-03-14

EPI_ISL_68305 EPI_ISL_84005 0.632736 2003-03-08 2003-03-13 2003-03-16

EPI_ISL_82420 EPI_ISL_68283 0.532040 2003-02-28 2003-03-06 2003-03-10

EPI_ISL_84005 EPI_ISL_82422 0.611640 2003-03-08 2003-03-12 2003-03-15

EPI_ISL_68290 EPI_ISL_68289 0.535616 2003-02-26 2003-03-06 2003-03-13

EPI_ISL_84006 EPI_ISL_82409 0.575564 2003-03-02 2003-03-06 2003-03-11

EPI_ISL_82421 EPI_ISL_82420 0.910588 2003-03-05 2003-03-11 2003-03-15

EPI_ISL_82422 EPI_ISL_68300 0.363844 2003-03-04 2003-03-08 2003-03-11

EPI_UNK_109 EPI_ISL_84011 0.135044 2003-03-05 2003-03-12 2003-03-15

EPI_ISL_82423 EPI_ISL_82415 0.151304 2003-03-10 2003-03-15 2003-03-19

EPI_ISL_82424 EPI_ISL_84008 0.376512 2003-03-08 2003-03-13 2003-03-17

EPI_ISL_84007 EPI_ISL_84023 0.622064 2003-03-09 2003-03-14 2003-03-18

EPI_ISL_82425 EPI_ISL_83997 0.181628 2003-03-09 2003-03-14 2003-03-18

EPI_ISL_84008 EPI_ISL_68291 0.587984 2003-03-08 2003-03-12 2003-03-16

EPI_ISL_84009 EPI_ISL_82414 0.243984 2003-03-04 2003-03-11 2003-03-15

EPI_ISL_82426 EPI_ISL_82395 0.626528 2003-03-05 2003-03-10 2003-03-15

EPI_ISL_68291 EPI_ISL_68289 0.532944 2003-03-04 2003-03-07 2003-03-10

EPI_ISL_82427 EPI_ISL_82407 0.654464 2003-03-03 2003-03-09 2003-03-13

EPI_ISL_84010 EPI_ISL_82417 0.713448 2003-03-08 2003-03-12 2003-03-16

EPI_UNK_120 EPI_ISL_82397 0.083712 2003-03-02 2003-03-09 2003-03-13

EPI_ISL_82428 EPI_ISL_82471 0.301992 2003-02-23 2003-02-28 2003-03-03

EPI_ISL_68336 EPI_ISL_83997 0.140388 2003-03-11 2003-03-15 2003-03-19

EPI_ISL_82429 EPI_ISL_82415 0.160396 2003-03-07 2003-03-13 2003-03-18

EPI_ISL_84011 EPI_ISL_82391 0.541288 2003-03-02 2003-03-06 2003-03-09

EPI_ISL_68337 EPI_ISL_84001 0.607340 2003-03-08 2003-03-13 2003-03-17

EPI_ISL_84012 EPI_ISL_83997 0.151696 2003-03-09 2003-03-15 2003-03-19

EPI_ISL_84013 EPI_ISL_82426 0.656604 2003-03-06 2003-03-13 2003-03-17

EPI_ISL_84014 EPI_ISL_83997 0.147472 2003-03-10 2003-03-15 2003-03-19

EPI_ISL_82430 EPI_ISL_82415 0.167220 2003-03-08 2003-03-14 2003-03-18

EPI_ISL_82431 EPI_ISL_82418 0.843452 2003-03-08 2003-03-14 2003-03-18

EPI_ISL_68312 EPI_ISL_68311 0.367008 2003-03-09 2003-03-15 2003-03-19

EPI_ISL_68292 EPI_ISL_82424 0.347896 2003-03-10 2003-03-15 2003-03-19

EPI_ISL_84015 EPI_ISL_83997 0.178980 2003-03-08 2003-03-13 2003-03-18

EPI_ISL_68293 EPI_ISL_68299 0.591812 2003-03-11 2003-03-15 2003-03-19

EPI_ISL_82432 EPI_ISL_68299 0.473856 2003-03-12 2003-03-16 2003-03-20

EPI_ISL_84016 EPI_ISL_83997 0.118660 2003-03-10 2003-03-16 2003-03-20

EPI_UNK_137 EPI_ISL_84011 0.097636 2003-03-09 2003-03-15 2003-03-19

EPI_ISL_68304 EPI_ISL_82415 0.156532 2003-03-09 2003-03-14 2003-03-19

EPI_ISL_84017 EPI_ISL_82425 0.115764 2003-03-11 2003-03-17 2003-03-21

EPI_ISL_68331 EPI_ISL_68330 0.692320 2003-03-11 2003-03-15 2003-03-19

EPI_ISL_68333 EPI_ISL_68332 0.975888 2003-03-11 2003-03-14 2003-03-18

EPI_UNK_142 EPI_ISL_82397 0.112920 2003-03-09 2003-03-15 2003-03-19

EPI_ISL_68294 EPI_ISL_68291 0.969172 2003-03-10 2003-03-14 2003-03-19

EPI_ISL_68334 EPI_ISL_68332 0.640408 2003-03-10 2003-03-14 2003-03-19

EPI_ISL_68323 EPI_ISL_84000 0.975728 2003-03-12 2003-03-17 2003-03-20

EPI_ISL_82433 EPI_ISL_82425 0.106204 2003-03-10 2003-03-16 2003-03-21

EPI_ISL_84018 EPI_ISL_82431 0.529260 2003-03-13 2003-03-18 2003-03-22

EPI_ISL_68352 EPI_ISL_82430 0.181828 2003-03-10 2003-03-16 2003-03-20

EPI_ISL_84019 EPI_ISL_84010 0.684596 2003-03-14 2003-03-18 2003-03-22

EPI_ISL_84020 EPI_ISL_82425 0.107916 2003-03-12 2003-03-18 2003-03-22

EPI_ISL_84021 EPI_ISL_83993 0.180636 2003-03-04 2003-03-12 2003-03-18

EPI_ISL_68335 EPI_ISL_68334 0.467212 2003-03-11 2003-03-16 2003-03-20

EPI_ISL_84022 EPI_ISL_82425 0.113312 2003-03-11 2003-03-17 2003-03-21

EPI_ISL_82434 EPI_ISL_68336 0.492724 2003-03-15 2003-03-20 2003-03-24

EPI_ISL_82435 EPI_ISL_84014 0.099336 2003-03-13 2003-03-19 2003-03-23

EPI_ISL_68307 EPI_ISL_84010 0.739120 2003-03-13 2003-03-18 2003-03-22

EPI_ISL_82436 EPI_ISL_82423 0.931160 2003-03-14 2003-03-20 2003-03-23

EPI_ISL_84023 EPI_ISL_68313 0.595488 2003-03-03 2003-03-09 2003-03-13

EPI_ISL_82437 EPI_ISL_82425 0.102856 2003-03-11 2003-03-18 2003-03-23

EPI_ISL_82438 EPI_ISL_68293 0.437732 2003-03-13 2003-03-19 2003-03-23

EPI_ISL_82439 EPI_ISL_68336 0.437452 2003-03-15 2003-03-20 2003-03-24

EPI_ISL_82440 EPI_ISL_84017 0.090600 2003-03-15 2003-03-21 2003-03-25

EPI_ISL_82441 EPI_ISL_82432 0.929592 2003-03-15 2003-03-21 2003-03-24

EPI_ISL_82442 EPI_ISL_68336 0.337860 2003-03-16 2003-03-21 2003-03-25

EPI_ISL_84024 EPI_ISL_84017 0.088044 2003-03-15 2003-03-21 2003-03-25

EPI_ISL_68295 EPI_ISL_68293 0.378728 2003-03-14 2003-03-20 2003-03-24

EPI_ISL_82443 EPI_ISL_84007 0.758072 2003-03-15 2003-03-20 2003-03-23

EPI_ISL_82444 EPI_ISL_84016 0.089532 2003-03-14 2003-03-20 2003-03-24

EPI_ISL_82445 EPI_ISL_84014 0.087744 2003-03-14 2003-03-20 2003-03-24

EPI_ISL_82446 EPI_ISL_68293 0.337408 2003-03-14 2003-03-21 2003-03-25

EPI_ISL_82447 EPI_ISL_68336 0.246340 2003-03-17 2003-03-22 2003-03-26

EPI_ISL_82448 EPI_ISL_84025 0.347500 2003-03-13 2003-03-20 2003-03-25

EPI_ISL_82449 EPI_ISL_82448 0.297560 2003-03-13 2003-03-20 2003-03-25

EPI_ISL_82450 EPI_ISL_84017 0.083372 2003-03-15 2003-03-21 2003-03-26

EPI_ISL_68338 EPI_ISL_83998 0.654644 2003-03-09 2003-03-18 2003-03-24

EPI_ISL_68314 EPI_ISL_84011 0.504380 2003-03-10 2003-03-19 2003-03-25

EPI_ISL_68340 EPI_ISL_68334 0.414696 2003-03-13 2003-03-19 2003-03-25

EPI_ISL_82451 EPI_ISL_84018 0.892760 2003-03-16 2003-03-23 2003-03-26

EPI_ISL_82452 EPI_ISL_68335 0.936416 2003-03-16 2003-03-22 2003-03-26

EPI_ISL_82453 EPI_ISL_68305 0.561852 2003-03-14 2003-03-19 2003-03-24

EPI_ISL_84025 EPI_ISL_84011 0.262624 2003-03-13 2003-03-20 2003-03-25

EPI_ISL_82454 EPI_ISL_82439 0.203784 2003-03-18 2003-03-24 2003-03-27

EPI_ISL_68296 EPI_ISL_84017 0.084500 2003-03-15 2003-03-21 2003-03-26

EPI_ISL_82455 EPI_ISL_68340 0.397140 2003-03-17 2003-03-22 2003-03-26

EPI_ISL_82456 EPI_ISL_84019 0.958480 2003-03-19 2003-03-24 2003-03-28

EPI_ISL_68341 EPI_ISL_68312 0.276892 2003-03-22 2003-03-26 2003-03-29

EPI_UNK_187 EPI_ISL_68312 0.173136 2003-03-16 2003-03-22 2003-03-26

EPI_ISL_82457 EPI_ISL_68336 0.383324 2003-03-16 2003-03-21 2003-03-25

EPI_ISL_84026 EPI_ISL_82424 0.324852 2003-03-10 2003-03-16 2003-03-20

EPI_ISL_68324 EPI_ISL_82443 0.549536 2003-03-19 2003-03-25 2003-03-29

EPI_ISL_68275 EPI_ISL_82443 0.413372 2003-03-21 2003-03-26 2003-03-30

EPI_ISL_82458 EPI_ISL_82397 0.361384 2003-03-17 2003-03-23 2003-03-28

EPI_ISL_82459 EPI_ISL_68275 0.230416 2003-03-23 2003-03-28 2003-03-31

EPI_ISL_68297 EPI_ISL_84024 0.136560 2003-03-21 2003-03-26 2003-03-31

EPI_ISL_68342 EPI_ISL_82455 0.917128 2003-03-21 2003-03-27 2003-03-31

EPI_ISL_68325 EPI_ISL_82443 0.315688 2003-03-21 2003-03-27 2003-03-31

EPI_ISL_82460 EPI_ISL_68275 0.258432 2003-03-26 2003-03-31 2003-04-03

EPI_ISL_84027 EPI_ISL_68297 0.137324 2003-03-21 2003-03-27 2003-04-02

EPI_ISL_68298 EPI_ISL_68297 0.221500 2003-03-22 2003-03-28 2003-04-02

EPI_ISL_84028 EPI_ISL_68297 0.173468 2003-03-21 2003-03-28 2003-04-02

EPI_ISL_68345 EPI_ISL_68341 0.976712 2003-03-30 2003-04-02 2003-04-05

EPI_ISL_68348 EPI_ISL_68275 0.236384 2003-03-25 2003-03-30 2003-04-02

EPI_ISL_68327 EPI_ISL_68316 0.176984 2003-03-28 2003-04-02 2003-04-06

EPI_ISL_68326 EPI_ISL_82458 0.741324 2003-03-21 2003-03-29 2003-04-03

EPI_ISL_68316 EPI_ISL_68275 0.237564 2003-03-25 2003-03-31 2003-04-04

EPI_ISL_82461 EPI_ISL_82458 0.539684 2003-03-18 2003-03-27 2003-04-02

EPI_ISL_68318 EPI_ISL_68316 0.192568 2003-03-27 2003-04-02 2003-04-07

EPI_ISL_68350 EPI_ISL_82454 0.205484 2003-03-22 2003-03-29 2003-04-04

EPI_ISL_84029 EPI_ISL_68316 0.230776 2003-03-28 2003-04-03 2003-04-08

EPI_ISL_68319 EPI_ISL_68312 0.303296 2003-03-19 2003-03-28 2003-04-05

EPI_ISL_82462 EPI_ISL_82460 0.946528 2003-03-31 2003-04-05 2003-04-08

EPI_ISL_84030 EPI_ISL_68316 0.205572 2003-03-30 2003-04-05 2003-04-08

EPI_ISL_68288 EPI_ISL_82459 0.503908 2003-03-21 2003-04-02 2003-04-06

EPI_ISL_68328 EPI_ISL_68316 0.170452 2003-03-27 2003-04-02 2003-04-07

EPI_UNK_215 EPI_ISL_82459 0.119448 2003-03-29 2003-04-05 2003-04-09

EPI_ISL_68349 EPI_ISL_68348 0.951108 2003-03-30 2003-04-04 2003-04-08

EPI_ISL_68276 EPI_ISL_82459 0.473876 2003-04-01 2003-04-05 2003-04-09

EPI_ISL_68343 EPI_ISL_68345 0.461448 2003-03-27 2003-04-07 2003-04-11

EPI_ISL_68320 EPI_ISL_68327 0.663708 2003-04-04 2003-04-08 2003-04-11

EPI_ISL_82463 EPI_ISL_68341 0.889564 2003-03-28 2003-04-03 2003-04-10

EPI_ISL_82464 EPI_ISL_84030 0.937892 2003-04-05 2003-04-10 2003-04-13

EPI_ISL_68344 EPI_ISL_68345 0.649856 2003-04-05 2003-04-09 2003-04-13

EPI_ISL_68277 EPI_ISL_68276 0.436596 2003-04-03 2003-04-09 2003-04-13

EPI_UNK_224 EPI_ISL_68320 0.106972 2003-04-03 2003-04-10 2003-04-14

EPI_ISL_68286 EPI_ISL_82459 0.176348 2003-04-04 2003-04-10 2003-04-15

EPI_ISL_82465 EPI_ISL_68286 0.518820 2003-04-07 2003-04-13 2003-04-16

EPI_ISL_68329 EPI_ISL_68320 0.442500 2003-04-05 2003-04-12 2003-04-16

EPI_ISL_68321 EPI_ISL_68320 0.520404 2003-04-07 2003-04-13 2003-04-17

EPI_ISL_82466 EPI_ISL_68276 0.311412 2003-04-04 2003-04-11 2003-04-15

EPI_ISL_82467 EPI_ISL_82465 0.732020 2003-04-09 2003-04-15 2003-04-18

EPI_ISL_68278 EPI_ISL_68276 0.321424 2003-04-03 2003-04-09 2003-04-13

EPI_ISL_68322 EPI_ISL_82462 0.412744 2003-04-06 2003-04-12 2003-04-17

EPI_ISL_68346 EPI_ISL_68344 0.405412 2003-04-08 2003-04-14 2003-04-19

EPI_ISL_68351 EPI_ISL_68320 0.393728 2003-04-06 2003-04-13 2003-04-18

EPI_ISL_82468 EPI_ISL_68344 0.337516 2003-04-09 2003-04-15 2003-04-20

EPI_ISL_82469 EPI_ISL_68346 0.286252 2003-04-10 2003-04-16 2003-04-20

EPI_ISL_84031 EPI_ISL_68321 0.664136 2003-04-13 2003-04-19 2003-04-23

EPI_ISL_68347 EPI_ISL_68343 0.428072 2003-04-15 2003-04-23 2003-04-29

EPI_UNK_239 EPI_ISL_68347 0.433204 2003-04-19 2003-04-27 2003-05-03

EPI_ISL_82472 EPI_ISL_68330 0.519936 2003-03-11 2003-03-16 2003-03-20

EPI_UNK_241 EPI_ISL_68343 0.103944 2003-04-03 2003-04-10 2003-04-14

| **host** | **infector** | **support** | **inf.times.2.5.** | **inf.times.50.** | **inf.times.97.5.** |
| --- | --- | --- | --- | --- | --- |
| EPI_ISL_82373 | EPI_ISL_82375 | 0.135176 | -13.7773 | -10.5578 | -7.74004 |
| EPI_ISL_82374 | EPI_ISL_82373 | 0.2636 | -9.94598 | -6.71854 | -3.64852 |
| EPI_ISL_83984 | EPI_ISL_82381 | 0.202264 | -11.9456 | -8.75282 | -5.54297 |
| EPI_ISL_83985 | EPI_ISL_83984 | 0.238464 | -9.11499 | -6.50516 | -4.1854 |
| EPI_ISL_67934 | EPI_ISL_82373 | 0.24836 | -10.0539 | -6.70847 | -3.762 |
| EPI_ISL_83986 | EPI_ISL_82375 | 0.101816 | -14.1392 | -10.1164 | -6.53731 |
| EPI_UNK_7 | EPI_ISL_83985 | 0.126144 | -7.71881 | -4.1816 | -1.93395 |
| EPI_ISL_82375 | index | 0.428296 | -17.8198 | -14.277 | -11.5133 |
| EPI_ISL_83988 | EPI_ISL_82381 | 0.484256 | -12.5307 | -8.60292 | -4.95949 |
| EPI_ISL_82376 | EPI_ISL_68269 | 0.496384 | -6.03081 | -3.12588 | -0.85873 |
| EPI_ISL_83989 | EPI_ISL_83988 | 0.606968 | -7.65088 | -4.81737 | -2.13487 |
| EPI_ISL_68268 | EPI_ISL_82373 | 0.208576 | -9.41922 | -5.43515 | -2.10231 |
| EPI_ISL_83990 | EPI_ISL_83986 | 0.31848 | -8.83441 | -5.08275 | -1.88179 |
| EPI_ISL_68269 | EPI_ISL_82373 | 0.143288 | -9.82559 | -6.69574 | -3.7744 |
| EPI_ISL_82471 | EPI_ISL_83984 | 0.144408 | -9.40243 | -6.49398 | -3.27733 |
| EPI_ISL_82377 | EPI_ISL_82380 | 0.47824 | -3.53033 | -0.9935 | 1.281369 |
| EPI_ISL_82378 | EPI_ISL_82406 | 0.658904 | -6.32648 | -3.31998 | -0.62636 |
| EPI_ISL_82379 | EPI_ISL_83985 | 0.656048 | -4.8513 | -2.28479 | 0.136115 |
| EPI_ISL_82380 | EPI_ISL_82471 | 0.226928 | -6.43611 | -3.6481 | -0.91965 |
| EPI_ISL_82381 | EPI_ISL_82375 | 0.26168 | -14.9402 | -11.4596 | -6.99326 |
| EPI_ISL_82382 | EPI_ISL_82374 | 0.162744 | -7.45122 | -2.86176 | 0.66807 |
| EPI_ISL_82383 | EPI_ISL_82374 | 0.125648 | -8.36441 | -4.53332 | -1.21957 |
| EPI_ISL_82384 | EPI_ISL_82385 | 0.41948 | -4.10697 | -1.12254 | 1.477521 |
| EPI_ISL_82385 | EPI_ISL_83986 | 0.159888 | -7.96963 | -4.17182 | -1.01993 |
| EPI_ISL_68270 | EPI_ISL_83986 | 0.269152 | -7.15121 | -2.73681 | 0.999468 |
| EPI_ISL_68279 | EPI_ISL_83984 | 0.730336 | -6.68974 | -3.58189 | -0.44745 |
| EPI_ISL_82386 | EPI_ISL_83986 | 0.333608 | -8.62722 | -4.15463 | 0.37059 |
| EPI_ISL_82387 | EPI_ISL_83986 | 0.302928 | -9.30705 | -5.22204 | -0.88175 |
| EPI_ISL_82388 | EPI_ISL_82471 | 0.435488 | -6.5585 | -2.93814 | 0.501037 |
| EPI_ISL_68280 | EPI_ISL_83989 | 0.439688 | -4.79038 | -2.02902 | 0.869776 |
| EPI_ISL_82389 | EPI_ISL_82405 | 0.147344 | -6.7129 | -1.86347 | 1.850456 |
| EPI_ISL_82390 | EPI_ISL_83990 | 0.96596 | -3.65685 | 0.403497 | 3.688539 |
| EPI_ISL_82391 | EPI_ISL_83985 | 0.322712 | -4.54733 | -1.26727 | 2.133079 |
| EPI_ISL_83991 | EPI_ISL_83984 | 0.542248 | -5.70204 | -2.19519 | 1.809591 |
| EPI_ISL_68281 | EPI_ISL_82383 | 0.626704 | -4.37724 | -0.7866 | 2.053154 |
| EPI_ISL_82392 | EPI_ISL_83985 | 0.45148 | -4.7948 | -1.76985 | 1.392712 |
| EPI_ISL_82393 | EPI_ISL_82471 | 0.407944 | -6.28581 | -2.46922 | 1.392285 |
| EPI_ISL_68289 | EPI_ISL_83986 | 0.137528 | -3.38789 | 1.352953 | 4.290886 |
| EPI_ISL_68300 | EPI_ISL_68303 | 0.286216 | -0.67807 | 2.74318 | 5.467533 |
| EPI_ISL_68282 | EPI_ISL_83985 | 0.56564 | -6.10334 | -3.34081 | 1.431878 |
| EPI_ISL_82394 | EPI_ISL_82405 | 0.157872 | -7.50506 | -2.54312 | 1.138163 |
| EPI_ISL_82395 | EPI_ISL_68270 | 0.87216 | -0.81266 | 2.546809 | 5.128731 |
| EPI_ISL_82396 | EPI_ISL_82382 | 0.15812 | -2.85313 | 1.510108 | 4.941571 |
| EPI_ISL_82397 | EPI_ISL_82379 | 0.986072 | 0.673101 | 4.149266 | 7.233254 |
| EPI_ISL_68306 | EPI_ISL_68268 | 0.13048 | -3.56576 | 1.112043 | 5.099019 |
| EPI_ISL_82398 | EPI_ISL_82406 | 0.934896 | -3.64798 | -0.52176 | 2.09565 |
| EPI_ISL_83992 | EPI_ISL_83989 | 0.4186 | -4.64206 | -1.74101 | 1.059089 |
| EPI_ISL_68308 | EPI_ISL_82382 | 0.136104 | -3.36593 | 1.198657 | 5.471341 |
| EPI_ISL_68310 | EPI_ISL_83991 | 0.684368 | 0.199993 | 3.536253 | 6.316138 |
| EPI_ISL_82399 | EPI_ISL_83992 | 0.476504 | -2.06957 | 2.381184 | 5.995566 |
| EPI_ISL_82400 | EPI_ISL_82398 | 0.74944 | -0.64661 | 3.3519 | 7.170378 |
| EPI_ISL_82401 | EPI_ISL_82380 | 0.419224 | -1.38979 | 2.658519 | 6.597995 |
| EPI_UNK_53 | EPI_ISL_83986 | 0.048344 | -4.82329 | 1.55231 | 6.648575 |
| EPI_ISL_82402 | EPI_ISL_68289 | 0.652128 | 3.4521 | 6.284645 | 9.082393 |
| EPI_ISL_68271 | EPI_ISL_83984 | 0.419544 | -5.36147 | 0.06379 | 6.965084 |
| EPI_ISL_68272 | EPI_ISL_84003 | 0.63308 | 2.699862 | 6.346687 | 9.475191 |
| EPI_ISL_68283 | EPI_ISL_68282 | 0.549064 | -3.44526 | 0.569743 | 4.857982 |
| EPI_ISL_82403 | EPI_ISL_83991 | 0.575128 | 0.494981 | 3.821373 | 6.754764 |
| EPI_ISL_82404 | EPI_ISL_82428 | 0.905648 | -0.11812 | 4.40449 | 8.129217 |
| EPI_ISL_82405 | EPI_ISL_82373 | 0.235376 | -9.65707 | -6.23408 | -2.95418 |
| EPI_ISL_82406 | EPI_ISL_83984 | 0.221056 | -9.3085 | -6.328 | -3.53795 |
| EPI_ISL_82407 | EPI_ISL_82391 | 0.83728 | 0.832488 | 3.964148 | 6.921082 |
| EPI_ISL_68301 | EPI_ISL_68300 | 0.411304 | 2.960757 | 6.084756 | 9.754829 |
| EPI_ISL_82408 | EPI_ISL_68289 | 0.376888 | 4.015622 | 7.043139 | 10.23476 |
| EPI_ISL_68284 | EPI_ISL_68309 | 0.501624 | -0.79058 | 4.088205 | 8.749973 |
| EPI_ISL_68309 | EPI_ISL_82378 | 0.640192 | -3.72834 | 0.342541 | 4.581154 |
| EPI_ISL_68273 | EPI_ISL_82396 | 0.159496 | -0.30263 | 4.029498 | 7.628845 |
| EPI_ISL_68313 | EPI_ISL_82392 | 0.463456 | 0.726638 | 4.846485 | 8.124317 |
| EPI_ISL_68330 | EPI_ISL_68300 | 0.261464 | 4.261734 | 7.471381 | 10.72064 |
| EPI_ISL_68302 | EPI_ISL_68300 | 0.348464 | 3.279015 | 6.429121 | 10.15708 |
| EPI_ISL_68311 | EPI_ISL_82403 | 0.397904 | 4.529712 | 8.226718 | 11.26521 |
| EPI_ISL_83993 | EPI_ISL_82396 | 0.220872 | 0.904735 | 5.21738 | 8.591417 |
| EPI_ISL_83994 | EPI_ISL_83989 | 0.967176 | 0.031526 | 4.219891 | 9.489446 |
| EPI_ISL_68285 | EPI_ISL_68272 | 0.49932 | 4.322864 | 8.898581 | 12.38894 |
| EPI_ISL_83995 | EPI_ISL_68300 | 0.251448 | 3.460616 | 6.854567 | 11.42129 |
| EPI_ISL_68274 | EPI_ISL_82375 | 0.842768 | -11.451 | -4.42063 | 3.633241 |
| EPI_UNK_77 | EPI_ISL_82386 | 0.093704 | -5.00704 | 0.130782 | 4.056106 |
| EPI_ISL_82409 | EPI_ISL_83985 | 0.567264 | -4.78941 | -2.06268 | 0.629418 |
| EPI_ISL_83996 | EPI_ISL_82385 | 0.41184 | -3.19218 | 0.884187 | 4.823851 |
| EPI_ISL_82410 | EPI_ISL_82385 | 0.357872 | -2.35345 | 2.775849 | 7.475229 |
| EPI_ISL_83997 | EPI_ISL_82402 | 0.296224 | 5.880111 | 9.288537 | 11.97836 |
| EPI_ISL_82411 | EPI_ISL_82392 | 0.348552 | -1.10051 | 2.261664 | 5.067776 |
| EPI_ISL_83998 | EPI_ISL_82409 | 0.584008 | 1.94607 | 5.868501 | 10.99026 |
| EPI_ISL_68303 | EPI_ISL_83984 | 0.21672 | -5.19965 | -0.1515 | 8.231697 |
| EPI_ISL_83999 | EPI_ISL_82411 | 0.96408 | 2.516382 | 7.133319 | 10.44739 |
| EPI_ISL_84000 | EPI_ISL_68313 | 0.40904 | 5.857119 | 9.87785 | 13.42935 |
| EPI_ISL_82412 | EPI_ISL_82403 | 0.330984 | 4.335217 | 8.550655 | 12.83396 |
| EPI_UNK_88 | EPI_ISL_84011 | 0.094528 | 7.55848 | 10.67722 | 13.95597 |
| EPI_ISL_82413 | EPI_ISL_82401 | 0.337192 | 1.695027 | 5.67995 | 9.321444 |
| EPI_ISL_68299 | EPI_ISL_82402 | 0.288272 | 6.141763 | 9.618813 | 12.57264 |
| EPI_ISL_82414 | EPI_ISL_83993 | 0.27936 | 4.743857 | 8.869875 | 12.84924 |
| EPI_ISL_82415 | EPI_ISL_68300 | 0.266328 | 3.27054 | 6.97533 | 10.93564 |
| EPI_ISL_84001 | EPI_ISL_84006 | 0.713088 | 6.382489 | 9.96143 | 12.84567 |
| EPI_ISL_68332 | EPI_ISL_68306 | 0.572256 | 4.224332 | 7.226043 | 9.888016 |
| EPI_ISL_82416 | EPI_ISL_83992 | 0.384656 | -0.33223 | 5.180256 | 10.53225 |
| EPI_ISL_82417 | EPI_ISL_68306 | 0.664112 | 3.127493 | 6.690796 | 10.65795 |
| EPI_ISL_84002 | EPI_ISL_68300 | 0.418728 | 2.996402 | 6.081729 | 9.533165 |
| EPI_ISL_84003 | EPI_ISL_82385 | 0.272672 | -1.98703 | 2.199464 | 5.907795 |
| EPI_ISL_82418 | EPI_ISL_82386 | 0.206712 | 2.337962 | 9.27649 | 13.40859 |
| EPI_ISL_82419 | EPI_ISL_82414 | 0.239144 | 6.644958 | 11.37778 | 15.31463 |
| EPI_ISL_84004 | EPI_ISL_82403 | 0.295504 | 4.494932 | 9.055921 | 13.31654 |
| EPI_ISL_68305 | EPI_ISL_84005 | 0.63152 | 11.27396 | 14.25202 | 16.70937 |
| EPI_ISL_82420 | EPI_ISL_68283 | 0.556184 | 0.72206 | 5.529376 | 9.864031 |
| EPI_ISL_84005 | EPI_ISL_82422 | 0.594728 | 8.083167 | 11.17089 | 14.06386 |
| EPI_ISL_68290 | EPI_ISL_68289 | 0.417928 | 1.028982 | 5.12504 | 12.87244 |
| EPI_ISL_84006 | EPI_ISL_82409 | 0.555928 | 2.249423 | 5.135002 | 8.620698 |
| EPI_ISL_82421 | EPI_ISL_82420 | 0.89168 | 5.902228 | 11.49431 | 15.78541 |
| EPI_ISL_82422 | EPI_ISL_68300 | 0.360928 | 3.415056 | 6.488842 | 9.947172 |
| EPI_UNK_109 | EPI_ISL_84011 | 0.206576 | 9.024825 | 11.7545 | 15.33802 |
| EPI_ISL_82423 | EPI_ISL_82415 | 0.141056 | 10.21583 | 14.35405 | 18.08484 |
| EPI_ISL_82424 | EPI_ISL_84008 | 0.362664 | 10.49514 | 13.95592 | 17.35578 |
| EPI_ISL_84007 | EPI_ISL_84023 | 0.610336 | 10.21769 | 14.65712 | 17.9413 |
| EPI_ISL_82425 | EPI_ISL_83997 | 0.174344 | 9.409824 | 13.2756 | 16.77637 |
| EPI_ISL_84008 | EPI_ISL_68291 | 0.563632 | 8.049377 | 10.94545 | 14.41237 |
| EPI_ISL_84009 | EPI_ISL_82414 | 0.23808 | 6.976106 | 11.6811 | 15.9903 |
| EPI_ISL_82426 | EPI_ISL_82395 | 0.619168 | 4.516565 | 9.17187 | 13.5447 |
| EPI_ISL_68291 | EPI_ISL_68289 | 0.525456 | 4.263283 | 6.646852 | 9.039122 |
| EPI_ISL_82427 | EPI_ISL_82407 | 0.644952 | 3.916513 | 9.059637 | 13.53153 |
| EPI_ISL_84010 | EPI_ISL_82417 | 0.7292 | 8.322179 | 12.52452 | 15.76152 |
| EPI_UNK_120 | EPI_ISL_82397 | 0.070184 | 5.146301 | 9.030157 | 12.41071 |
| EPI_ISL_82428 | EPI_ISL_82471 | 0.298072 | -5.07654 | -0.66296 | 3.07537 |
| EPI_ISL_68336 | EPI_ISL_82408 | 0.127512 | 10.01737 | 14.41298 | 17.79647 |
| EPI_ISL_82429 | EPI_ISL_82415 | 0.168744 | 8.292384 | 12.92134 | 17.20347 |
| EPI_ISL_84011 | EPI_ISL_82391 | 0.479312 | 2.040666 | 5.587839 | 8.532882 |
| EPI_ISL_68337 | EPI_ISL_84001 | 0.586728 | 8.778398 | 13.42901 | 17.59314 |
| EPI_ISL_84012 | EPI_ISL_83997 | 0.14244 | 9.730983 | 13.90602 | 17.58233 |
| EPI_ISL_84013 | EPI_ISL_82426 | 0.642496 | 8.580562 | 13.67134 | 17.80741 |
| EPI_ISL_84014 | EPI_ISL_83997 | 0.141568 | 9.630522 | 13.6405 | 17.39739 |
| EPI_ISL_82430 | EPI_ISL_82415 | 0.167088 | 8.943338 | 13.41047 | 17.51987 |
| EPI_ISL_82431 | EPI_ISL_82418 | 0.821592 | 9.842933 | 14.48644 | 18.08266 |
| EPI_ISL_68312 | EPI_ISL_68311 | 0.384416 | 9.52239 | 14.67735 | 18.74926 |
| EPI_ISL_68292 | EPI_ISL_82424 | 0.33636 | 11.52153 | 15.98905 | 19.82601 |
| EPI_ISL_84015 | EPI_ISL_82408 | 0.170864 | 8.177348 | 12.68424 | 17.6658 |
| EPI_ISL_68293 | EPI_ISL_68299 | 0.572368 | 10.74743 | 14.16166 | 17.81691 |
| EPI_ISL_82432 | EPI_ISL_68299 | 0.425784 | 11.17315 | 14.53649 | 18.25519 |
| EPI_ISL_84016 | EPI_ISL_83997 | 0.111144 | 9.765404 | 14.16581 | 18.29898 |
| EPI_UNK_137 | EPI_ISL_84011 | 0.100696 | 9.593133 | 14.12897 | 17.97971 |
| EPI_ISL_68304 | EPI_ISL_82415 | 0.16232 | 8.77505 | 13.75896 | 18.13909 |
| EPI_ISL_84017 | EPI_ISL_82425 | 0.114024 | 12.71555 | 16.9532 | 20.64342 |
| EPI_ISL_68331 | EPI_ISL_68330 | 0.690208 | 10.33017 | 14.32697 | 18.07603 |
| EPI_ISL_68333 | EPI_ISL_68332 | 0.980576 | 11.58578 | 14.60256 | 18.32246 |
| EPI_UNK_142 | EPI_ISL_84011 | 0.11116 | 9.168226 | 13.85751 | 18.38075 |
| EPI_ISL_68294 | EPI_ISL_68291 | 0.97756 | 10.71236 | 14.28593 | 18.96174 |
| EPI_ISL_68334 | EPI_ISL_68332 | 0.635432 | 9.352099 | 12.75677 | 17.30066 |
| EPI_ISL_68323 | EPI_ISL_84000 | 0.973072 | 11.9369 | 16.67924 | 20.14541 |
| EPI_ISL_82433 | EPI_ISL_82425 | 0.102272 | 12.06437 | 16.57583 | 20.50618 |
| EPI_ISL_84018 | EPI_ISL_82431 | 0.51496 | 14.14573 | 18.38647 | 21.77965 |
| EPI_ISL_68352 | EPI_ISL_82430 | 0.172688 | 12.26119 | 16.78779 | 20.93258 |
| EPI_ISL_84019 | EPI_ISL_84010 | 0.649152 | 13.62409 | 17.65406 | 21.18833 |
| EPI_ISL_84020 | EPI_ISL_82425 | 0.1038 | 13.02063 | 17.7209 | 21.83847 |
| EPI_ISL_84021 | EPI_ISL_82414 | 0.17228 | 7.374391 | 12.37337 | 17.28384 |
| EPI_ISL_68335 | EPI_ISL_68334 | 0.456928 | 12.96077 | 16.6086 | 20.06815 |
| EPI_ISL_84022 | EPI_ISL_82425 | 0.105432 | 12.75479 | 17.00622 | 20.99917 |
| EPI_ISL_82434 | EPI_ISL_68336 | 0.46884 | 15.42577 | 19.14792 | 22.72864 |
| EPI_ISL_82435 | EPI_ISL_84014 | 0.094568 | 13.97291 | 18.77033 | 22.60054 |
| EPI_ISL_68307 | EPI_ISL_84010 | 0.7312 | 12.97253 | 17.42854 | 22.04853 |
| EPI_ISL_82436 | EPI_ISL_82423 | 0.925296 | 15.49009 | 19.93933 | 23.47604 |
| EPI_ISL_84023 | EPI_ISL_68313 | 0.5756 | 5.164646 | 9.154211 | 12.67736 |
| EPI_ISL_82437 | EPI_ISL_82425 | 0.102528 | 12.95437 | 17.54269 | 21.95879 |
| EPI_ISL_82438 | EPI_ISL_68293 | 0.404704 | 14.41272 | 18.9434 | 22.91805 |
| EPI_ISL_82439 | EPI_ISL_68336 | 0.4256 | 15.87523 | 19.4069 | 23.07081 |
| EPI_ISL_82440 | EPI_ISL_84017 | 0.089664 | 16.29693 | 21.07747 | 24.66025 |
| EPI_ISL_82441 | EPI_ISL_82432 | 0.921376 | 16.88337 | 20.87144 | 24.44598 |
| EPI_ISL_82442 | EPI_ISL_68336 | 0.326112 | 15.74834 | 19.72156 | 23.63379 |
| EPI_ISL_84024 | EPI_ISL_84017 | 0.089688 | 15.91707 | 21.1974 | 24.559 |
| EPI_ISL_68295 | EPI_ISL_68293 | 0.368272 | 14.58227 | 19.43565 | 23.70756 |
| EPI_ISL_82443 | EPI_ISL_84007 | 0.740408 | 15.95258 | 20.07022 | 23.06642 |
| EPI_ISL_82444 | EPI_ISL_84017 | 0.08876 | 15.43616 | 20.43905 | 24.16918 |
| EPI_ISL_82445 | EPI_ISL_84012 | 0.087776 | 14.6595 | 19.52855 | 24.02407 |
| EPI_ISL_82446 | EPI_ISL_68293 | 0.314328 | 14.45358 | 19.6874 | 24.49726 |
| EPI_ISL_82447 | EPI_ISL_68336 | 0.24936 | 16.15413 | 20.10052 | 24.4055 |
| EPI_ISL_82448 | EPI_ISL_84025 | 0.350656 | 16.43733 | 21.27933 | 25.5312 |
| EPI_ISL_82449 | EPI_ISL_82448 | 0.284136 | 16.43733 | 21.62981 | 25.96974 |
| EPI_ISL_82450 | EPI_ISL_84017 | 0.083656 | 16.12907 | 21.37183 | 25.5521 |
| EPI_ISL_68338 | EPI_ISL_83998 | 0.628752 | 8.028064 | 15.48834 | 23.92032 |
| EPI_ISL_68314 | EPI_ISL_84011 | 0.486016 | 9.091913 | 14.24534 | 23.4143 |
| EPI_ISL_68340 | EPI_ISL_68334 | 0.410488 | 13.45488 | 18.5373 | 23.48629 |
| EPI_ISL_82451 | EPI_ISL_84018 | 0.883512 | 18.37846 | 22.91311 | 26.40145 |
| EPI_ISL_82452 | EPI_ISL_68335 | 0.928536 | 16.69219 | 21.68586 | 25.78926 |
| EPI_ISL_82453 | EPI_ISL_68305 | 0.551216 | 14.45665 | 19.22362 | 24.17687 |
| EPI_ISL_84025 | EPI_ISL_84011 | 0.258256 | 11.63136 | 15.91393 | 20.65501 |
| EPI_ISL_82454 | EPI_ISL_82439 | 0.19736 | 19.4972 | 24.02673 | 27.63389 |
| EPI_ISL_68296 | EPI_ISL_84017 | 0.085328 | 16.03094 | 21.37539 | 25.86553 |
| EPI_ISL_82455 | EPI_ISL_68340 | 0.415784 | 17.28344 | 22.4494 | 26.07336 |
| EPI_ISL_82456 | EPI_ISL_84019 | 0.952112 | 18.75504 | 23.61138 | 27.77372 |
| EPI_ISL_68341 | EPI_ISL_68319 | 0.283832 | 22.80544 | 26.34929 | 29.46394 |
| EPI_UNK_187 | EPI_ISL_68312 | 0.163728 | 16.74672 | 21.12438 | 25.46516 |
| EPI_ISL_82457 | EPI_ISL_68336 | 0.373416 | 15.68905 | 19.51102 | 23.56527 |
| EPI_ISL_84026 | EPI_ISL_82424 | 0.330712 | 11.58616 | 16.51818 | 20.53298 |
| EPI_ISL_68324 | EPI_ISL_82443 | 0.499368 | 20.43417 | 24.31961 | 27.84109 |
| EPI_ISL_68275 | EPI_ISL_82443 | 0.396336 | 21.15192 | 25.00766 | 28.68186 |
| EPI_ISL_82458 | EPI_ISL_82397 | 0.445896 | 15.53038 | 22.20392 | 26.9558 |
| EPI_ISL_82459 | EPI_ISL_68275 | 0.229872 | 25.02648 | 28.65411 | 31.32257 |
| EPI_ISL_68297 | EPI_ISL_82440 | 0.130656 | 21.87255 | 26.47702 | 30.51258 |
| EPI_ISL_68342 | EPI_ISL_82455 | 0.9062 | 21.21668 | 26.66417 | 31.17758 |
| EPI_ISL_68325 | EPI_ISL_82443 | 0.31216 | 20.8357 | 25.138 | 29.95027 |
| EPI_ISL_82460 | EPI_ISL_68275 | 0.245176 | 26.55011 | 30.43319 | 33.76465 |
| EPI_ISL_84027 | EPI_ISL_68297 | 0.146376 | 24.52952 | 29.09368 | 33.15945 |
| EPI_ISL_68298 | EPI_ISL_68297 | 0.219328 | 25.38747 | 29.84747 | 33.79236 |
| EPI_ISL_84028 | EPI_ISL_68297 | 0.174456 | 25.15773 | 29.51225 | 33.4104 |
| EPI_ISL_68345 | EPI_ISL_68341 | 0.981808 | 30.41061 | 33.30791 | 36.14545 |
| EPI_ISL_68348 | EPI_ISL_68275 | 0.228632 | 25.64932 | 29.44478 | 32.93578 |
| EPI_ISL_68327 | EPI_ISL_68316 | 0.164368 | 30.32877 | 33.76324 | 36.87799 |
| EPI_ISL_68326 | EPI_ISL_82458 | 0.725056 | 22.65007 | 28.8437 | 34.41632 |
| EPI_ISL_68316 | EPI_ISL_68275 | 0.235624 | 26.25799 | 30.63164 | 34.67516 |
| EPI_ISL_82461 | EPI_ISL_82458 | 0.563896 | 21.35427 | 27.15435 | 32.82702 |
| EPI_ISL_68318 | EPI_ISL_68316 | 0.18924 | 29.78555 | 34.37207 | 38.00382 |
| EPI_ISL_68350 | EPI_ISL_82454 | 0.210536 | 23.3883 | 28.84092 | 34.81794 |
| EPI_ISL_84029 | EPI_ISL_68316 | 0.235432 | 30.27445 | 35.05354 | 39.15202 |
| EPI_ISL_68319 | EPI_ISL_68312 | 0.282576 | 16.74073 | 22.44807 | 34.53947 |
| EPI_ISL_82462 | EPI_ISL_82460 | 0.943592 | 32.40812 | 36.41991 | 39.38351 |
| EPI_ISL_84030 | EPI_ISL_68316 | 0.200024 | 31.44094 | 35.76592 | 39.41169 |
| EPI_ISL_68288 | EPI_ISL_82459 | 0.499904 | 29.76917 | 33.50848 | 36.70035 |
| EPI_ISL_68328 | EPI_ISL_68316 | 0.168312 | 29.53757 | 34.04321 | 38.16479 |
| EPI_UNK_215 | EPI_ISL_82459 | 0.121192 | 30.3474 | 34.10917 | 38.82123 |
| EPI_ISL_68349 | EPI_ISL_68348 | 0.947664 | 30.53979 | 35.01376 | 39.41707 |
| EPI_ISL_68276 | EPI_ISL_82459 | 0.470704 | 30.88396 | 34.79773 | 38.34659 |
| EPI_ISL_68343 | EPI_ISL_68341 | 0.453024 | 26.80165 | 31.25077 | 38.81029 |
| EPI_ISL_68320 | EPI_ISL_68327 | 0.618952 | 34.9111 | 38.58363 | 41.91698 |
| EPI_ISL_82463 | EPI_ISL_68341 | 0.887472 | 27.61131 | 32.406 | 39.78309 |
| EPI_ISL_82464 | EPI_ISL_84030 | 0.928584 | 36.22745 | 41.27487 | 44.85644 |
| EPI_ISL_68344 | EPI_ISL_68345 | 0.699936 | 35.55237 | 39.80516 | 43.33697 |
| EPI_ISL_68277 | EPI_ISL_68276 | 0.418984 | 35.41073 | 39.86838 | 43.72522 |
| EPI_UNK_224 | EPI_ISL_68320 | 0.08572 | 38.21346 | 41.8496 | 45.40328 |
| EPI_ISL_68286 | EPI_ISL_68276 | 0.176904 | 35.46033 | 39.2344 | 43.5031 |
| EPI_ISL_82465 | EPI_ISL_68286 | 0.50708 | 39.92661 | 43.56924 | 46.52181 |
| EPI_ISL_68329 | EPI_ISL_68320 | 0.43116 | 38.49487 | 42.48422 | 47.1242 |
| EPI_ISL_68321 | EPI_ISL_68320 | 0.511344 | 39.66082 | 43.91102 | 47.56588 |
| EPI_ISL_82466 | EPI_ISL_68276 | 0.312528 | 35.75053 | 41.05347 | 46.12598 |
| EPI_ISL_82467 | EPI_ISL_82465 | 0.722704 | 42.64317 | 46.27033 | 49.23041 |
| EPI_ISL_68278 | EPI_ISL_68276 | 0.31352 | 35.26448 | 39.41393 | 43.68804 |
| EPI_ISL_68322 | EPI_ISL_82462 | 0.38428 | 37.4331 | 41.92206 | 47.69173 |
| EPI_ISL_68346 | EPI_ISL_68344 | 0.41896 | 40.23599 | 44.71858 | 48.71198 |
| EPI_ISL_68351 | EPI_ISL_68320 | 0.381416 | 38.87812 | 43.12802 | 48.31799 |
| EPI_ISL_82468 | EPI_ISL_68344 | 0.350432 | 40.27839 | 44.98681 | 49.31802 |
| EPI_ISL_82469 | EPI_ISL_68346 | 0.289928 | 43.70867 | 48.05858 | 51.67247 |
| EPI_ISL_84031 | EPI_ISL_68321 | 0.663024 | 44.70732 | 50.39514 | 54.8378 |
| EPI_ISL_68347 | EPI_ISL_82469 | 0.3848 | 48.65639 | 54.57054 | 59.82217 |
| EPI_UNK_239 | EPI_ISL_68347 | 0.363576 | 52.80859 | 59.72289 | 64.78651 |
| EPI_ISL_82472 | EPI_ISL_68330 | 0.53792 | 10.5223 | 14.54276 | 18.86767 |
| EPI_UNK_241 | EPI_ISL_68320 | 0.092232 | 38.08087 | 41.91955 | 45.23061 |
