# Supplementary material for: Simultaneous inference of phylogenetic and transmission trees in infectious disease outbreaks
Source: PLoS Comput Biol. 2017 May 18;13(5):e1005495. doi: 10.1371/journal.pcbi.1005495 (PMC5436636; doi:10.1371/journal.pcbi.1005495)
Supplement: S1 Methods — (DOCX) [file pcbi.1005495.s002.docx]

**S2 Methods**

**Part 1: The posterior probability of the transmission tree and phylogenetic tree.**

Definitions:

- *n* = outbreak size
- **S** with elements *S_i_* = sampling times per host $i\in\left[1,n\right]$
- **G** with elements *G_i_* = genome sequences per host $i\in\left[1,n\right]$, each consisting of *L* nucleotides *A*, *C*, *G*, *T*, or – if unknown – *N*
  - $D_{ij}$ = number of different nucleotides between hosts *i* and *j*, not counting *N*’s
  - $D_{\max}$ = maximum $D_{ij}$ for all host pairs *i* and *j*
  - $N_{ij}$ = number of nucleotide positions with *N* in host *i* and/or *j*
  - $d_{ij}=D_{ij}+N_{ij}D_{\max}/L$ = sequence distance between hosts *i* and *j*
- **I** with elements *I_i_* = infection times per host $i\in\left[1,n\right]$
- **M** with elements *M_i_* = infectors per host $i\in\left[1,n\right]$
- *P* is the phylogenetic tree, being a set of numbered nodes *x*:
  - $x\in\left[1,n\right]$ are the sampling nodes, corresponding to hosts *i*
  - $x\in\left[n+1,2n-1\right]$ are the coalescent nodes
  - $x\in\left[2n,3n-1\right]$ are the transmission nodes, with node *x* corresponding to the infection of host $i=x+1-2n$
- Further, we define *t_x_* as the time of node *x*, *v_x_* as the ancestor node of node *x*, and *h_x_* as the host in which node *x* resides (transmission nodes assigned to the infector, as the value of *x* already defines the infectee)
- $mrca_{x,y}$ = MRCA (most recent common ancestor) of nodes *x* and *y*
- *P_i_* are the phylogenetic trees within each host: $P_{i}=\left\{x\left|h_{x}=i\right.\right\}$, for which we further define $\tau_{x}=t_{x}-t_{h_{x}+2n-1}$ as the time of the node since infection of the host.
- **θ** = parameter vector, with parameters for generation time and sampling time distributions, the parameters describing the within-host coalescent model, and the mutation rate
  - $a_{G},m_{G}$ = shape parameter and mean of gamma-distributed generation time
  - $a_{S},m_{S}$ = shape parameter and mean of gamma-distributed sampling time
  - *r* = slope of within-host pathogen growth model
  - $\mu$ = mutation rate

*notation*

- $d_{\Gamma\left(a,m\right)};p_{\Gamma\left(a,m\right)}$ are the density and cumulative density of a Gamma distribution with shape parameter *a* and mean *m*
- $\Pr\left(.\right)$ is a probability or probability density
- $u\left(\tau\right)$ is the heaviside step function, equal to 0 if $\tau<0$, and equal to 1 if $\tau\geq0$

The complete posterior probability

$$\begin{matrix} \Pr\left(\mathbf{I},\mathbf{M},P,\mathbf{\theta}\left|\mathbf{S},\mathbf{G}\right.\right)\propto\Pr\left(\mathbf{S},\mathbf{G}\left|\mathbf{I},\mathbf{M},P,\mathbf{\theta}\right.\right)\cdot\Pr\left(\mathbf{I},\mathbf{M},P,\mathbf{\theta}\right) \\ =\Pr\left(\mathbf{S},\mathbf{G}\left|\mathbf{I},\mathbf{M},P,\mathbf{\theta}\right.\right)\cdot\Pr\left(\mathbf{I},\mathbf{M},P\left|\mathbf{\theta}\right.\right)\cdot\Pr\left(\mathbf{\theta}\right) \\ =\Pr\left(\mathbf{S},\mathbf{G},\mathbf{I},\mathbf{M},P\left|\mathbf{\theta}\right.\right)\cdot\Pr\left(\mathbf{\theta}\right) \\ =\Pr\left(\mathbf{G}\left|\mathbf{S},\mathbf{I},\mathbf{M},P,\mathbf{\theta}\right.\right)\cdot\Pr\left(P\left|\mathbf{S},\mathbf{I},\mathbf{M},\mathbf{\theta}\right.\right)\cdot\Pr\left(\mathbf{S}\left|\mathbf{I},\mathbf{M},\mathbf{\theta}\right.\right)\cdot\Pr\left(\mathbf{I},\mathbf{M}\left|\mathbf{\theta}\right.\right)\cdot\Pr\left(\mathbf{\theta}\right) \\ =\Pr\left(\mathbf{G}\left|P\right.,\mathbf{\theta}\right)\cdot\Pr\left(P\left|\mathbf{S},\mathbf{I},\mathbf{M},\mathbf{\theta}\right.\right)\cdot\Pr\left(\mathbf{S}\left|\mathbf{I},\mathbf{\theta}\right.\right)\cdot\Pr\left(\mathbf{I},\mathbf{M}\left|\mathbf{\theta}\right.\right)\cdot\Pr\left(\mathbf{\theta}\right) \end{matrix}$$

All steps until the last follow from standard probability rules. In the last step, dependencies are removed that do not exist in the model. In the last line, the first four terms are likelihood terms, that will be elaborated below. The last term is the prior.

Likelihood #1 for the genetic data

We assume a Jukes-Cantor substitution model with mutation rate *μ*:

For each locus, for each possible assignment at each internal node (coalescent and transmission), for all branches in the tree indicated by the end node *x*, the probability of a mutation is calculated where $I_{mut}$ indicates if a mutation occurred on that branch and N indicates if the branch ends in a tip without observed nucleotide (‘n’ in the sequence data). This likelihood can be calculated using Felsenstein’s pruning algorithm [1]. Note that in the above formulation the actual rate of nucleotide *change* is 0.75*µ*, as a mutation gives rise to any of the four nucleotides.

Likelihood #2 for the phylogenetic tree

The likelihood for the complete phylogenetic tree is a product of the likelihoods of trees in individual hosts:

$$\Pr\left(P\left|\mathbf{S},\mathbf{I},\mathbf{M},\mathbf{\theta}\right.\right)=\prod_{i} \Pr\left(P_{i}\left|S_{i},\mathbf{I},\mathbf{M},\mathbf{\theta}\right.\right)$$

The dependency on the complete vectors **I** and **M** remains, because these determine the transmission nodes with host *i* as infector.

We define the number of lineages in host *i* at time *τ* since infection as

$L_{i}\left(\tau\right)=1+\sum_{x\left|x\in P_{i}\cap n<x<2n\right.} u\left(\tau-\tau_{x}\right)-\sum_{x\left|x\in P_{i}\cap x\geq2n\right.} u\left(\tau-\tau_{x}\right)-u\left(\tau-\tau_{i}\right)$ ,

where $u\left(\tau\right)$ is the heaviside step function, i.e. $u\left(\tau\right)=0$ if $\tau<0$ and $u\left(\tau\right)=1$ if $\tau\geq0$, adding 1 at infection and at each coalescent node, and subtracting 1 at each transmission node and at sampling .

The within-host dynamics $w\left(\tau,r\right)$ describes the product of pathogen generation time and effective population size, the inverse of which determines the coalescent rate. As a general form, we choose

$w\left(\tau,r\right)=r\tau$,

which automatically gives a bottleneck of size 1 (because the coalescent rate $\to\infty$ as $\tau\downarrow0$), allows for most coalescent nodes close to the time of infection (high *r*) or close to the transmission nodes (low *r*).

Thus, the likelihood for the phylogenetic tree in host *i* becomes

$\Pr\left(P_{i}\left|S_{i},\mathbf{I},\mathbf{M},\mathbf{\theta}\right.\right)=\exp\left(-\int_{0}^{\infty} \left(\begin{matrix} L_{i}\left(\tau\right) \\ 2 \end{matrix}\right)\frac{1}{w\left(\tau,r\right)}d\tau\right)\prod_{x\left|x\in P_{i}\cap n<x<2n\right.} \frac{1}{w\left(\tau_{x},r\right)}$ ,

with $\left(\begin{matrix} 0 \\ 2 \end{matrix}\right)\equiv\left(\begin{matrix} 1 \\ 2 \end{matrix}\right)\equiv0$.

Likelihood #3 for the sampling intervals

The sampling intervals are assumed to follow a gamma distribution with shape parameter *a_S_* and with mean $m_{S}$. The likelihood is the product of densities for all sampling times:

$$\Pr\left(\mathbf{S}\left|\mathbf{I},\mathbf{\theta}\right.\right)=\prod_{i} d_{\Gamma\left(a_{S},m_{S}\right)}\left(S_{i}-I_{i}\right)$$

Likelihood #4 for infection times and infectors

The generation intervals are assumed to follow a gamma distribution with shape parameter *a_G_* and with mean $m_{G}$. The likelihood is the product of densities for all generation intervals multiplied by the probability of each transmission tree topology which we leave out because we assume that every topology is a priori equally likely:

$$\Pr\left(\mathbf{I},\mathbf{M}\left|\mathbf{\theta}\right.\right)=\Pr\left(\mathbf{I}\left|\mathbf{M},\mathbf{\theta}\right.\right)\cdot\Pr\left(\mathbf{M}\left|\mathbf{\theta}\right.\right)=\prod_{i\left|M_{i}>0\right.} d_{\Gamma\left(a_{G},m_{G}\right)}\left(I_{i}-I_{M_{i}}\right)$$

Prior distributions

The model has 6 parameters: mutation rate *μ*, within-host growth rate *r*, sampling interval distribution parameters *a_S_* and *m_S_*, and generation interval distribution parameters *a_G_* and *m_G_*. In the current implementation, *a_S_* and *a_G_* are not estimated but chosen before the analysis. The prior distributions for the other parameters are

- $\log\left(\mu\right)\sim U\left(-\infty,+\infty\right)$, which corresponds to a relative density on the original scale of $d\left(\mu\right)\propto1/\mu$.
- $m_{S}\sim D\left(\mu_{0,S},\sigma_{0,S}\right)$, a prior distribution with mean $\mu_{0,S}$ and standard deviation $\sigma_{0,S}$, which is translated (see box below) into a Gamma-distributed prior for the rate parameter *b_S_* of the sampling interval Gamma distribution: $b_{S}\sim\Gamma\left(a_{0,S}=2+\mu_{0,S}^{2}/\sigma_{0,S}^{2},b_{0,S}=\left(\mu_{0,S}/a_{S}\right)\cdot\left(1+\mu_{0,S}^{2}/\sigma_{0,S}^{2}\right)\right)$. Here, $a_{0,S}$ is the shape and $b_{0,S}$ is the rate. When using an uninformative prior, we set $\mu_{0,S}=1$ and $\sigma_{0,S}=\infty$.
- $m_{G}\sim D\left(\mu_{0,G},\sigma_{0,G}\right)$, as for *m_S_*.
- $r\sim\Gamma\left(a_{0,r},m_{0,r}\right)$. When using an uninformative prior, we set $a_{0,r}=3$ and $m_{0,r}=1$.

$$\begin{matrix} \text{We have }a_{S}\text{ fixed, and define }b_{S}\sim\Gamma\left(a_{0,S},b_{0,S}\right)\text{, such that} \\ \text{1. }E\left(a_{S}/b_{S}\right)=\mu_{0,S}\Leftrightarrow E\left(1/b_{S}\right)=\mu_{0,S}/a_{S} \\ \text{2. }E\left(\left(\mu_{0,S}-a_{S}/b_{S}\right)^{2}\right)=\sigma_{0,S}^{2}\Leftrightarrow E\left(\left(\mu_{0,S}/a_{S}-1/b_{S}\right)^{2}\right)=\sigma_{0,S}^{2}/a_{S}^{2} \\ \text{Now, we wish to calculate }a_{0,S}\text{ and }b_{0,S}. \\ \text{If the density of rate }b_{S}\text{ is }d_{\Gamma}\left(b_{S}\right)\text{, then the density of scale }\theta_{S}=1/b_{S}\text{ is }d_{\Gamma}\left(\theta_{S}\right)/\theta_{S}^{2}\text{. This is an inverse} \\ \text{Gamma distribution, of which the mean and variance can be filled into the equations above:} \\ \text{1. }\mu_{0,S}/a_{S}=\frac{b_{0,S}}{a_{0,S}-1} \\ \text{2. }\sigma_{0,S}^{2}/a_{S}^{2}=\frac{b_{0,S}^{2}}{\left(a_{0,S}-1\right)^{2}\left(a_{0,S}-2\right)} \\ \text{Solving these equations for }a_{0,S}\text{ and }b_{0,S}\text{ gives the prior distribution for }b_{S}. \end{matrix}$$

**Part 2: Updating steps in the MCMC chain.**

The mean sampling and generation intervals are directly sampled from their posteriors. For the other parameters in **θ** and unobserved variables **Z** = {**I, M,** *P*}, updating is always done by a Metropolis-Hastings step:

- proposing new values with proposal density $G\left(\mathbf{\theta}'\left|\mathbf{\theta}\right.\right)$ or $H\left(\mathbf{Z}'\left|\mathbf{Z}\right.,\mathbf{S},\mathbf{\theta}\right)$, respectively
- accepting the new values with probability *α*,

$$\alpha=\min\left[1,\frac{\Pr\left(\mathbf{S},\mathbf{G},\mathbf{Z}\left|\mathbf{\theta}'\right.\right)\cdot\Pr\left(\mathbf{\theta}'\right)\cdot G\left(\mathbf{\theta}\left|\mathbf{\theta}'\right.\right)}{\Pr\left(\mathbf{S},\mathbf{G},\mathbf{Z}\left|\mathbf{\theta}\right.\right)\cdot\Pr\left(\mathbf{\theta}\right)\cdot G\left(\mathbf{\theta}'\left|\mathbf{\theta}\right.\right)}\right]$$

or

$\alpha=\min\left[1,\frac{\Pr\left(\mathbf{S},\mathbf{G},\mathbf{Z}'\left|\mathbf{\theta}\right.\right)\cdot\Pr\left(\mathbf{\theta}\right)\cdot H\left(\mathbf{Z}\left|\mathbf{Z}',\mathbf{S},\mathbf{\theta}\right.\right)}{\Pr\left(\mathbf{S},\mathbf{G},\mathbf{Z}\left|\mathbf{\theta}\right.\right)\cdot\Pr\left(\mathbf{\theta}\right)\cdot H\left(\mathbf{Z}'\left|\mathbf{Z},\mathbf{S},\mathbf{\theta}\right.\right)}\right]=\min\left[1,\frac{\Pr\left(\mathbf{S},\mathbf{G},\mathbf{Z}'\left|\mathbf{\theta}\right.\right)\cdot H\left(\mathbf{Z}\left|\mathbf{Z}'\right.,\mathbf{S},\mathbf{\theta}\right)}{\Pr\left(\mathbf{S},\mathbf{G},\mathbf{Z}\left|\mathbf{\theta}\right.\right)\cdot H\left(\mathbf{Z}'\left|\mathbf{Z}\right.,\mathbf{S},\mathbf{\theta}\right)}\right]$,

respectively

Updating the mean sampling and generation intervals

The parameters $m_{S}$ and $m_{G}$ are updated by direct sampling from the posterior distributions of rate parameters $b_{S}$ and $b_{G}$ and calculating $m_{S}=a_{S}/b_{S}$ and $m_{G}=a_{G}/b_{G}$. These posterior distributions are:

$$\begin{matrix} b_{S}\sim\Gamma\left(a_{post,S}=a_{0,S}+n\cdot a_{S},b_{post,S}=b_{0,S}+\sum_{i} S_{i}-I_{i}\right) \\ b_{G}\sim\Gamma\left(a_{post,G}=a_{0,G}+\left(n-1\right)\cdot a_{G},b_{post,G}=b_{0,G}+\sum_{i\left|M_{i}\neq0\right.} I_{i}-I_{M_{i}}\right) \end{matrix}$$

Updating other parameters in **θ**

*Proposal*

A new value $\zeta'$ (being $\mu'$ or $r'$) is proposed as

$$\log\left(\zeta'\right)\sim N\left(\log\left(\zeta\right),\sigma_{\zeta}^{2}\right)$$

Thus, the proposal density of $\zeta'$ is

$$G\left(\zeta'\left|\zeta\right.\right)=d_{N}\left(\log\left(\zeta'\right)-\log\left(\zeta\right),\sigma_{\zeta}^{2}\right)/\zeta'$$

The proposal variance $\sigma_{\zeta}^{2}$ is calculated from the available data as follows:

- For $\log\left(\mu\right)$, the posterior density is proportional to the likelihood (because of the uniform prior). Although the actual likelihood calculation is done with Felsenstein’s pruning algorithm [1], *μ* is the rate of a Poisson process, so it should be possible to express it in terms of a number of realizations and the total exposure time. The number of realizations *x* is the number of mutations, i.e. the parsimony of the phylogenetic tree; the exposure time *D* is the sum of all branch length multiplied by the sequence length. By expressing the likelihood of $\log\left(\mu\right)$ in terms of *x* and *D*, and normalizing it to get a proper distribution, the variance of this distribution can be calculated as $\psi_{1}\left(x\right)$, which is the trigamma function. Optimal proposals in a Metropolis-Hastings step have a variance of 2.38^2^ times the target variance [2]. Assuming that the posterior phylogenetic trees reach maximum parsimony, the proposal variance is set to $\sigma_{\mu}^{2}=2.38^{2}\psi_{1}\left(x\right)$, with *x* the number of SNPs in the dataset.
- For $\log\left(r\right)$, a similar reasoning is used (although the prior distribution is different), because $1/r$ is proportional to the rate of a Poisson process as well. Here, the number of realizations *x* is the number of coalescent nodes, which is equal to *n* – 1. So, $\sigma_{r}^{2}=2.38^{2}\psi_{1}\left(n-1\right)$.

*Acceptance probability*

The acceptance probability is

$$\begin{matrix} \min\left[1,\frac{\Pr\left(\mathbf{S},\mathbf{G},\mathbf{Z}\left|\mathbf{\theta}'\right.\right)\cdot\Pr\left(\mathbf{\theta}'\right)\cdot G\left(\mathbf{\theta}\left|\mathbf{\theta}'\right.\right)}{\Pr\left(\mathbf{S},\mathbf{G},\mathbf{Z}\left|\mathbf{\theta}\right.\right)\cdot\Pr\left(\mathbf{\theta}\right)\cdot G\left(\mathbf{\theta}'\left|\mathbf{\theta}\right.\right)}\right] \\ =\min\left[1,\frac{\Pr\left(\mathbf{S},\mathbf{G},\mathbf{Z}\left|\mathbf{\theta}'\right.\right)\cdot\Pr\left(\zeta'\right)\cdot d_{N}\left(\log\left(\zeta\right)-\log\left(\zeta'\right),\sigma_{\zeta}^{2}\right)/\zeta}{\Pr\left(\mathbf{S},\mathbf{G},\mathbf{Z}\left|\mathbf{\theta}\right.\right)\cdot\Pr\left(\zeta\right)\cdot d_{N}\left(\log\left(\zeta'\right)-\log\left(\zeta\right),\sigma_{\zeta}^{2}\right)/\zeta'}\right] \end{matrix}$$

For *μ*, the prior and proposal densities cancel out, and we are left with the acceptance probability $\min\left[1,\Pr\left(\mathbf{G}\left|P,\mu'\right.\right)/\Pr\left(\mathbf{G}\left|P,\mu\right.\right)\right]$. For *r*, the acceptance probability becomes

$$\min\left[1,\frac{\Pr\left(P\left|\mathbf{S},\mathbf{I},\mathbf{M},r'\right.\right)d_{\Gamma\left(a_{0,r},m_{0,r}\right)}\left(r'\right)/r}{\Pr\left(P\left|\mathbf{S},\mathbf{I},\mathbf{M},r\right.\right)d_{\Gamma\left(a_{0,r},m_{0,r}\right)}\left(r\right)/r'}\right]$$

Updating the transmission tree and phylogenetic tree

*Proposals*

The unobserved variables are updated in small sets at once. Three types of proposal are distinguished: the first in which both transmission tree and phylogenetic tree are changed, the second in which only the transmission tree is changed, and the third in which only the phylogenetic tree is changed. In the R package the user can choose the probabilities with which these three proposals are used; the default is 80%-20%-0% for the three types, respectively.

All three proposals take a single focal host *i*; proposals 1 and 2 start with step 1:

1. propose a new infection time $I_{i}'$ by sampling $T\sim\Gamma\left(a_{P},m_{S}\right)$ from a Gamma distribution with shape = and mean = $m_{S}$, and calculating $I_{i}'=S_{i}-T$

Then, the following decision trees determine the subsequent proposal steps and acceptance probabilities, laid out in proposal paths A-K below. In some cases, the proposal is immediately rejected if no reverse proposal exists:

Proposal 1: changing phylogenetic and transmission trees.

Q1: is host *i* index case?

{Q1=Y}Q2: is $I_{i}'<\min\left(\left\{I_{j}\left|M_{j}=i\right.\right\}\right)$ → is $I_{i}'$ before host *i*’s first transmission node?

{Q12=YY} **follow proposal path A (Fig 5A)**

{Q12=YN}Q3: is $I_{i}'<\left\{I_{j}\left|M_{j}=i\right.\right\}_{\left(2\right)}$ → is $I_{i}'$ before host *i*’s second transmission node?

(Yes, if no second transmission node exists)

{Q123=YNY} **follow proposal path B (Fig 5B)**

{Q123=YNN} **follow proposal path C (Fig 5C)**

{Q1=N}Q2: is $I_{i}'<\min\left(\mathbf{I}\right)$ → is $I_{i}'$ before infection of the index case?

{Q12=NY} **follow proposal path D (Fig 5D)**

{Q12=NN}Q3: is $I_{i}'<\min\left(\left\{I_{j}\left|M_{j}=i\right.\right\}\right)$ → is $I_{i}'$ before host *i*’s first transmission node?

(Yes, if no transmission node exists)

{Q123=NNY} **follow proposal path E (Fig 5E)**

{Q123=NNN} **follow proposal path F (Fig 5F)**

Proposal 2: changing transmission tree, but not phylogenetic tree.

Q1: is host *i* index case?

{Q1=Y}Q2: is $I_{i}'<\min\left(\left\{\tau_{x}\left|h_{x}=i\right.\right\}\right)$ → is $I_{i}'$ before host *i*’s first coalescent node?

{Q12=YY} **follow proposal path G**

{Q12=YN} **reject**

{Q1=N}Q2: is $I_{i}'>mrca_{i,M_{i}}$ → is $I_{i}'$ after the *mrca* of the samplings in hosts *i* and $M_{i}$?

{Q12=NY} **follow proposal path H (Fig 6A)**

{Q12=NN}: new proposal step:

1. propose $I_{M_{i}}'$ by sampling $T\sim\Gamma\left(a_{P},m_{S}\right)$ and calculating $I_{M_{i}}'=S_{M_{i}}-T$

Q3: is $I_{M_{i}}'>mrca_{i,M_{i}}$→ is $I_{M_{i}}'$ after the *mrca* of host *i* and its infector?

{Q123=NNY}Q4: is $M_{M_{i}}=0$ → is host *i*'s infector the index case?

{Q1234=NNYY} **follow proposal path I (Fig 6B)**

{Q1234=NNYN}Q5: is $I_{i}'$ after the *mrca* of the samplings in hosts *i* and $M_{M_{i}}$?

{Q12345=NNYNY} **follow proposal path J (Fig 6C)**

{Q12345=NNYNN} **reject**

{Q123=NNN} **reject**

Proposal 3: changing phylogenetic tree topology only.

**follow proposal path K**

**Proposal path A {Tree 1, Q12=YY}**

*Situation*

Host *i* is the index case, and $I_{i}'$ is before its first transmission node.

*Proposed changes to transmission tree*

1. Topological changes: none
2. Infection time changes:
   1. $I_{i}$ changes to $I_{i}'$

*Proposal steps*

1. propose a new tree $P_{i}'$ by simulating the within-host coalescent model

*Proposal distribution*

The proposal distribution for $I_{i}'$ and $P_{i}'$ is

*Acceptance probability*

The acceptance probability (removing the dependency on **θ** after the first line, for readability) is

$$\begin{matrix} \min\left[1,\frac{\Pr\left(\mathbf{S},\mathbf{G},\mathbf{Z}'\left|\mathbf{\theta}\right.\right)\times H\left(\mathbf{Z}\left|\mathbf{Z}',\mathbf{S},\mathbf{\theta}\right.\right)}{\Pr\left(\mathbf{S},\mathbf{G},\mathbf{Z}\left|\mathbf{\theta}\right.\right)\times H\left(\mathbf{Z}'\left|\mathbf{Z},\mathbf{S},\mathbf{\theta}\right.\right)}\right] \\ =\min\left[1,\frac{\Pr\left(\mathbf{G}\left|P'\right.\right)\cdot\Pr\left(P'\left|\mathbf{S},\mathbf{I}',\mathbf{M}\right.\right)\cdot\Pr\left(\mathbf{S}\left|\mathbf{I}'\right.\right)\cdot\Pr\left(\mathbf{I}',\mathbf{M}\right)\times\Pr\left(P_{i}\left|S_{i},\mathbf{I},\mathbf{M}\right.\right)\cdot d_{\Gamma\left(a_{P},m_{S}\right)}\left(S_{i}-I_{i}\right)}{\Pr\left(\mathbf{G}\left|P\right.\right)\cdot\Pr\left(P\left|\mathbf{S},\mathbf{I},\mathbf{M}\right.\right)\cdot\Pr\left(\mathbf{S}\left|\mathbf{I}\right.\right)\cdot\Pr\left(\mathbf{I},\mathbf{M}\right)\times\Pr\left(P_{i}'\left|S_{i},\mathbf{I}',\mathbf{M}\right.\right)\cdot d_{\Gamma\left(a_{P},m_{S}\right)}\left(S_{i}-I_{i}'\right)}\right] \\ =\min\left[1,\frac{\Pr\left(\mathbf{G}\left|P'\right.\right)\cdot\Pr\left(S_{i}\left|I_{i}'\right.\right)\cdot\Pr\left(\mathbf{I}',\mathbf{M}\right)\cdot d_{\Gamma\left(a_{P},m_{S}\right)}\left(S_{i}-I_{i}\right)}{\Pr\left(\mathbf{G}\left|P\right.\right)\cdot\Pr\left(S_{i}\left|I_{i}\right.\right)\cdot\Pr\left(\mathbf{I},\mathbf{M}\right)\cdot d_{\Gamma\left(a_{P},m_{S}\right)}\left(S_{i}-I_{i}'\right)}\right] \end{matrix}$$

*Reverse proposal*

This sampling step can be reversed by proposing through (the same) proposal path A, with the original infection time *I_i_* proposed for the same focal host *i* (going back in Fig 5A).

**Proposal path B {Tree 1, Q123=YNY}**

*Situation*

Host *i* is index case and $I_{i}'$ is sampled after its first transmission node (infecting host *j*), but before its second transmission node, if there is any.

*Proposed changes to transmission tree*

1. Topological changes:
   1. host *i* gets a new infector
   2. host *j* becomes index case
2. Infection time change:
   1. $I_{i}$ changes to $I_{i}'$

*Proposal steps*

1. propose a new infector $M_{i}'$ from the proposal distribution $F_{j}\left(I_{i}'\right)$, which is the probability to select infector *j* at time $I_{i}'$ in the outbreak. The proposal distribution is given by

$F_{j}\left(t\right)=\frac{u\left(t-I_{j}\right)\cdot\left(d_{\Gamma\left(a_{G},b_{G}\right)}\left(t-I_{j}\right)+1/d_{ij}\right)}{\sum_{k} \left[u\left(t-I_{k}\right)\cdot\left(d_{\Gamma\left(a_{G},b_{G}\right)}\left(t-I_{k}\right)+1/d_{ij}\right)\right]}$ ,

so the probability to select infector *j* is proportional to the density of the generation time plus a weight $1/d_{ij}$, with $d_{ij}$ equal to the sequence distance between isolates from hosts *i* and *j* (see list of definitions). This proposal distribution gives a sampling weight to possible infectors based on their infection time and related to the genetic distance between isolates.

1. host *j* becomes the index case: $M_{j}'=0$
2. bookkeeping: make corresponding changes in the nodes and move one of the coalescent nodes from host *i* to host $M_{i}'$
3. propose new trees $P_{i}'$ and $P_{M_{i}'}'$ by simulating the within-host coalescent model

*Proposal distribution*

The proposal distribution for $I_{i}'$, $M_{i}'$, $M_{j}'$, $P_{i}'$, and $P_{M_{i}'}'$ is

Here, $\Pr\left(M_{j}'\left|...\right.\right)=1$ because it follows automatically from the proposed $I_{i}'$.

*Acceptance probability*

The acceptance probability (removing the dependency on **θ** after the first line, for readability) is

$$\begin{matrix} \min\left[1,\frac{\Pr\left(\mathbf{S},\mathbf{G},\mathbf{Z}'\left|\mathbf{\theta}\right.\right)\times H\left(\mathbf{Z}\left|\mathbf{Z}',\mathbf{S},\mathbf{\theta}\right.\right)}{\Pr\left(\mathbf{S},\mathbf{G},\mathbf{Z}\left|\mathbf{\theta}\right.\right)\times H\left(\mathbf{Z}'\left|\mathbf{Z},\mathbf{S},\mathbf{\theta}\right.\right)}\right] \\ =\min\left[1,\frac{\Pr\left(\mathbf{G}\left|P'\right.\right)\cdot\Pr\left(P'\left|\mathbf{S},\mathbf{I}',\mathbf{M}'\right.\right)\cdot\Pr\left(\mathbf{S}\left|\mathbf{I}'\right.\right)\cdot\Pr\left(\mathbf{I}',\mathbf{M}'\right)\times\Pr\left(P_{i},P_{M_{i}'}\left|\mathbf{S},\mathbf{I},\mathbf{M}\right.\right)\cdot d_{\Gamma\left(a_{P},m_{S}\right)}\left(S_{i}-I_{i}\right)}{\Pr\left(\mathbf{G}\left|P\right.\right)\cdot\Pr\left(P\left|\mathbf{S},\mathbf{I},\mathbf{M}\right.\right)\cdot\Pr\left(\mathbf{S}\left|\mathbf{I}\right.\right)\cdot\Pr\left(\mathbf{I},\mathbf{M}\right)\times\Pr\left(P_{i}',P_{M_{i}'}'\left|\mathbf{S},\mathbf{I}',\mathbf{M}'\right.\right)\cdot F_{M_{i}'}\left(I_{i}'\right)\cdot d_{\Gamma\left(a_{P},m_{S}\right)}\left(S_{i}-I_{i}'\right)}\right] \\ =\min\left[1,\frac{\Pr\left(\mathbf{G}\left|P'\right.\right)\cdot\Pr\left(S_{i}\left|I_{i}'\right.\right)\cdot\Pr\left(\mathbf{I}',\mathbf{M}'\right)\cdot d_{\Gamma\left(a_{P},m_{S}\right)}\left(S_{i}-I_{i}\right)}{\Pr\left(\mathbf{G}\left|P\right.\right)\cdot\Pr\left(S_{i}\left|I_{i}\right.\right)\cdot\Pr\left(\mathbf{I},\mathbf{M}\right)\cdot F_{M_{i}'}\left(I_{i}'\right)\cdot d_{\Gamma\left(a_{P},m_{S}\right)}\left(S_{i}-I_{i}'\right)}\right] \end{matrix}$$

*Reversal*

Reversal is possible through proposal path D. Fig 5D shows the reverse proposal path D from the original path B in Fig 5B.

**Proposal path C {Tree 1, Q123=YNN}**

*Situation*

Host *i* is the index case and $I_{i}'$ is sampled after its second transmission node. Host *j* is the first secondary case.

*Proposed changes to transmission tree*

1. Topological changes:
   1. host *j* becomes index case, transmitting to host *i*
   2. with 50% probability: the other secondary cases of hosts *i* and *j* are exchanged
2. Infection time change:
   1. the sampled $I_{i}'$ is discarded; instead, the infection times of hosts *i* and *j* are switched

*Proposal steps*

1. discard the sampled infection time and switch infection times by proposing $I_{i}'=I_{j}$ and $I_{j}'=I_{i}$
2. switch role by proposing $M_{i}'=j$ and $M_{j}'=0$
3. bookkeeping: make corresponding changes in the nodes and move one of the coalescent nodes from host *i* to host *j*
4. with 50% probability: for all $\left\{k\left|M_{k}=i\wedge k\neq j\right.\right\}$, propose $M_{k}'=j$; for all $\left\{k\left|M_{k}=j\right.\right\}$, propose $M_{k}'=i$; finish by bookkeeping: swap all transmission and coalescent nodes between *P_i_* and *P_j_*
5. propose new trees $P_{i}'$ and $P_{j}'$ by simulating the within-host coalescent model

*Proposal distribution*

The proposal distribution for $I_{i}'$, $I_{j}'$, **M**, $P_{i}'$, and $P_{j}'$ is

Here, $\Pr\left(\mathbf{M}'\left|\mathbf{M},\mathbf{\theta}\right.\right)=0.5$ because of the two possible rearrangements of infectees that follow automatically from the proposed $I_{i}'$, and $\Pr\left(I_{j}'\left|I_{i}',\mathbf{\theta}\right.\right)=1$ because it follows automatically from the proposed $I_{i}'$. In the last step, $\Pr\left(I_{i}'\left|\mathbf{I},S_{i},\mathbf{\theta}\right.\right)=p_{\Gamma\left(a_{P},b_{S}\right)}\left(S_{i}-I_{j}\right)$ is the cumulative density of the sampling interval distribution, which is the probability of taking proposal path C.

*Acceptance probability*

The acceptance probability (removing the dependency on **θ** after the first line, for readability) is

$$\begin{matrix} \min\left[1,\frac{\Pr\left(\mathbf{S},\mathbf{G},\mathbf{Z}'\left|\mathbf{\theta}\right.\right)\times H\left(\mathbf{Z}\left|\mathbf{Z}',\mathbf{S},\mathbf{\theta}\right.\right)}{\Pr\left(\mathbf{S},\mathbf{G},\mathbf{Z}\left|\mathbf{\theta}\right.\right)\times H\left(\mathbf{Z}'\left|\mathbf{Z},\mathbf{S},\mathbf{\theta}\right.\right)}\right] \\ =\min\left[1,\frac{\Pr\left(\mathbf{G}\left|P'\right.\right)\cdot\Pr\left(P'\left|\mathbf{S},\mathbf{I}',\mathbf{M}'\right.\right)\cdot\Pr\left(\mathbf{S}\left|\mathbf{I}'\right.\right)\cdot\Pr\left(\mathbf{I}',\mathbf{M}'\right)\times\Pr\left(P_{i},P_{j}\left|\mathbf{S},\mathbf{I},\mathbf{M}\right.\right)\cdot0.5\cdot p_{\Gamma\left(a_{P},m_{S}\right)}\left(S_{j}-I_{j}\right)}{\Pr\left(\mathbf{G}\left|P\right.\right)\cdot\Pr\left(P\left|\mathbf{S},\mathbf{I},\mathbf{M}\right.\right)\cdot\Pr\left(\mathbf{S}\left|\mathbf{I}\right.\right)\cdot\Pr\left(\mathbf{I},\mathbf{M}\right)\times\Pr\left(P_{i}',P_{j}'\left|\mathbf{S},\mathbf{I}',\mathbf{M}'\right.\right)\cdot0.5\cdot p_{\Gamma\left(a_{P},m_{S}\right)}\left(S_{i}-I_{j}\right)}\right] \\ =\min\left[1,\frac{\Pr\left(\mathbf{G}\left|P'\right.\right)\cdot\Pr\left(\mathbf{S}\left|\mathbf{I}'\right.\right)\cdot\Pr\left(\mathbf{I}',\mathbf{M}'\right)\cdot p_{\Gamma\left(a_{P},m_{S}\right)}\left(S_{j}-I_{j}\right)}{\Pr\left(\mathbf{G}\left|P\right.\right)\cdot\Pr\left(\mathbf{S}\left|\mathbf{I}\right.\right)\cdot\Pr\left(\mathbf{I},\mathbf{M}\right)\cdot p_{\Gamma\left(a_{P},m_{S}\right)}\left(S_{i}-I_{j}\right)}\right] \end{matrix}$$

*Reversal*

Reversal is possible through proposal path C, with focal host *j*. In Fig 5C, reversal takes place by proposing an infection time for host IV after its second transmission event. It is important to note that if host IV would not have a second transmission event, the reversal step is impossible; therefore, proposal path C is always rejected if the proposed new index ends up with only one secondary case.

**Proposal path D {Tree 1, Q123=NY}**

*Situation*

Host *i* is not the index case, and $I_{i}'$ is sampled before infection of the index case

*Proposed changes to transmission tree*

1. Topological changes:
   1. host *i* becomes index case, and the original index case *j* its first secondary case
2. Infection time change:
   1. $I_{i}$ changes to $I_{i}'$

*Proposal steps*

1. host *i* becomes the index case: $M_{i}'=0$
2. host *i* becomes the infector of the original index case: $M_{j}'=i$
3. bookkeeping: make corresponding changes in the nodes and move one of the coalescent nodes from host $M_{i}$ to host *i*
4. propose new trees $P_{i}'$ and $P_{M_{i}}'$ by simulating the within-host coalescent model

*Proposal distribution*

The proposal distribution for $I_{i}'$, $M_{i}'$, $M_{j}'$, $P_{i}'$, and $P_{M_{i}}'$ is

Here, $\Pr\left(M_{i}',M_{j}'\left|...\right.\right)=1$ because it follows automatically from the proposed $I_{i}'$.

*Acceptance probability*

The acceptance probability (removing the dependency on **θ** after the first line, for readability) is

$$\begin{matrix} \min\left[1,\frac{\Pr\left(\mathbf{S},\mathbf{G},\mathbf{Z}'\left|\mathbf{\theta}\right.\right)\times H\left(\mathbf{Z}\left|\mathbf{Z}',\mathbf{S},\mathbf{\theta}\right.\right)}{\Pr\left(\mathbf{S},\mathbf{G},\mathbf{Z}\left|\mathbf{\theta}\right.\right)\times H\left(\mathbf{Z}'\left|\mathbf{Z},\mathbf{S},\mathbf{\theta}\right.\right)}\right] \\ =\min\left[1,\frac{\Pr\left(\mathbf{G}\left|P'\right.\right)\cdot\Pr\left(P'\left|\mathbf{S},\mathbf{I}',\mathbf{M}'\right.\right)\cdot\Pr\left(\mathbf{S}\left|\mathbf{I}'\right.\right)\cdot\Pr\left(\mathbf{I}',\mathbf{M}'\right)\times\Pr\left(P_{i},P_{M_{i}}\left|\mathbf{S},\mathbf{I},\mathbf{M}\right.\right)\cdot F_{M_{i}}\left(I_{i}\right)\cdot d_{\Gamma\left(a_{P},m_{S}\right)}\left(S_{i}-I_{i}\right)}{\Pr\left(\mathbf{G}\left|P\right.\right)\cdot\Pr\left(P\left|\mathbf{S},\mathbf{I},\mathbf{M}\right.\right)\cdot\Pr\left(\mathbf{S}\left|\mathbf{I}\right.\right)\cdot\Pr\left(\mathbf{I},\mathbf{M}\right)\times\Pr\left(P_{i}',P_{M_{i}}'\left|\mathbf{S},\mathbf{I}',\mathbf{M}'\right.\right)\cdot d_{\Gamma\left(a_{P},m_{S}\right)}\left(S_{i}-I_{i}'\right)}\right] \\ =\min\left[1,\frac{\Pr\left(\mathbf{G}\left|P'\right.\right)\cdot\Pr\left(S_{i}\left|I_{i}'\right.\right)\cdot\Pr\left(\mathbf{I}',\mathbf{M}'\right)\cdot F_{M_{i}}\left(I_{i}\right)\cdot d_{\Gamma\left(a_{P},m_{S}\right)}\left(S_{i}-I_{i}\right)}{\Pr\left(\mathbf{G}\left|P\right.\right)\cdot\Pr\left(S_{i}\left|I_{i}\right.\right)\cdot\Pr\left(\mathbf{I},\mathbf{M}\right)\cdot d_{\Gamma\left(a_{P},m_{S}\right)}\left(S_{i}-I_{i}'\right)}\right] \end{matrix}$$

Here, $F_{M_{i}}\left(I_{i}\right)$ is the probability of proposing the original infector in the reverse proposal (path B).

*Reversal*

Reversal is possible through proposal path B. Fig 5B shows the reverse proposal path B from the original path D in Fig 5D.

**Proposal path E {Tree 1, Q123=NNY}**

*Situation*

Host *i* is not the index case, and $I_{i}'$ is sampled after infection of the index case, but before the first transmission node of host *i*, if there is any.

*Proposed changes to transmission tree*

1. Topological changes:
   1. host *i* gets a (possibly) new infector
2. Infection time changes:
   1. $I_{i}$ changes to $I_{i}'$

*Proposal steps*

1. propose a new infector $M_{i}'$ from the proposal distribution $F_{j}\left(I_{i}'\right)$, which is the probability to select infector *j* at time $I_{i}'$ in the outbreak. The proposal distribution is given by

$F_{j}\left(t\right)=\frac{u\left(t-I_{j}\right)\cdot\left(d_{\Gamma\left(a_{G},b_{G}\right)}\left(t-I_{j}\right)+1/d_{ij}\right)}{\sum_{k} \left[u\left(t-I_{k}\right)\cdot\left(d_{\Gamma\left(a_{G},b_{G}\right)}\left(t-I_{k}\right)+1/d_{ij}\right)\right]}$ ,

so the probability to select infector *j* is proportional to the density of the generation time plus a weight $1/d_{ij}$, with $d_{ij}$ equal to the sequence distance between isolates from hosts *i* and *j* (see list of definitions). This proposal distribution gives a sampling weight to possible infectors based on their infection time and related to the genetic distance between isolates.

1. bookkeeping: make corresponding changes in the nodes and move one of the coalescent nodes from host $M_{i}$ to host $M_{i}'$
2. propose new trees $P_{i}'$, $P_{M_{i}}'$, and $P_{M_{i}'}'$ by simulating the within-host coalescent model

*Proposal distribution*

The proposal distribution for $I_{i}'$, $M_{i}'$, $P_{i}'$, $P_{M_{i}}'$, and $P_{M_{i}'}'$ is

*Acceptance probability*

The acceptance probability (removing the dependency on **θ** after the first line, for readability) is

$$\begin{matrix} \min\left[1,\frac{\Pr\left(\mathbf{S},\mathbf{G},\mathbf{Z}'\left|\mathbf{\theta}\right.\right)\times H\left(\mathbf{Z}\left|\mathbf{Z}',\mathbf{S},\mathbf{\theta}\right.\right)}{\Pr\left(\mathbf{S},\mathbf{G},\mathbf{Z}\left|\mathbf{\theta}\right.\right)\times H\left(\mathbf{Z}'\left|\mathbf{Z},\mathbf{S},\mathbf{\theta}\right.\right)}\right] \\ =\min\left[1,\frac{\Pr\left(\mathbf{G}\left|P'\right.\right)\cdot\Pr\left(P'\left|\mathbf{S},\mathbf{I}',\mathbf{M}'\right.\right)\cdot\Pr\left(\mathbf{S}\left|\mathbf{I}'\right.\right)\cdot\Pr\left(\mathbf{I}',\mathbf{M}'\right)\times\Pr\left(P_{i},P_{M_{i}},P_{M_{i}'}\left|\mathbf{S},\mathbf{I},\mathbf{M}\right.\right)\cdot F_{M_{i}}\left(I_{i}\right)\cdot d_{\Gamma\left(a_{P},m_{S}\right)}\left(S_{i}-I_{i}\right)}{\Pr\left(\mathbf{G}\left|P\right.\right)\cdot\Pr\left(P\left|\mathbf{S},\mathbf{I},\mathbf{M}\right.\right)\cdot\Pr\left(\mathbf{S}\left|\mathbf{I}\right.\right)\cdot\Pr\left(\mathbf{I},\mathbf{M}\right)\times\Pr\left(P_{i}',P_{M_{i}}',P_{M_{i}'}'\left|\mathbf{S},\mathbf{I}',\mathbf{M}'\right.\right)\cdot F_{M_{i}'}\left(I_{i}'\right)\cdot d_{\Gamma\left(a_{P},m_{S}\right)}\left(S_{i}-I_{i}'\right)}\right] \\ =\min\left[1,\frac{\Pr\left(\mathbf{G}\left|P'\right.\right)\cdot\Pr\left(S_{i}\left|I_{i}'\right.\right)\cdot\Pr\left(\mathbf{I}',\mathbf{M}'\right)\cdot F_{M_{i}}\left(I_{i}\right)\cdot d_{\Gamma\left(a_{P},m_{S}\right)}\left(S_{i}-I_{i}\right)}{\Pr\left(\mathbf{G}\left|P\right.\right)\cdot\Pr\left(S_{i}\left|I_{i}\right.\right)\cdot\Pr\left(\mathbf{I},\mathbf{M}\right)\cdot F_{M_{i}'}\left(I_{i}'\right)\cdot d_{\Gamma\left(a_{P},m_{S}\right)}\left(S_{i}-I_{i}'\right)}\right] \end{matrix}$$

*Reversal*

Reversal is possible through proposal path E, with the original infection time *I_i_* proposed for the same focal host *i* (going back in Fig 5E).

**Proposal path F {Tree 1, Q12=NNY}**

*Situation*

Host *i* is not the index case and $I_{i}'$ is sampled after infection of its first infectee *j*

*Proposed changes to transmission tree*

1. Topological changes:
   1. $M_{i}$ becomes infector of host *j*
   2. host *j* becomes infector of host *i*
   3. with 50% probability: the other secondary cases of hosts *i* and *j* are exchanged
2. Infection time change:
   1. the sampled $I_{i}'$ is discarded; instead, the infection times of hosts *i* and *j* are switched

*Proposal steps*

1. discard the sampled infection time and switch infection times by proposing $I_{i}'=I_{j}$ and $I_{j}'=I_{i}$
2. switch role by proposing $M_{i}'=j$ and $M_{j}'=M_{i}$
3. bookkeeping: make corresponding changes in the nodes and move one of the coalescent nodes from host *i* to host *j*
4. with 50% probability: for all $\left\{k\left|M_{k}=i\wedge k\neq j\right.\right\}$, propose $M_{k}'=j$; for all $\left\{k\left|M_{k}=j\right.\right\}$, propose $M_{k}'=i$; finish by bookkeeping: swap all transmission and coalescent nodes between *P_i_* and *P_j_*
5. propose new trees $P_{i}'$ and $P_{j}'$ by simulating the within-host coalescent model

*Proposal distribution*

The proposal distribution for $I_{i}'$, $I_{j}'$, $M_{i}'$, $M_{j}'$, $P_{i}'$, and $P_{j}'$ is

Here, $\Pr\left(\mathbf{M}'\left|\mathbf{I}',\mathbf{\theta}\right.\right)=0.5$ because of the two possible rearrangements of infectees that follow automatically from the proposed $I_{i}'$, and $\Pr\left(I_{j}'\left|I_{i}',\mathbf{\theta}\right.\right)=1$ because it follows automatically from the proposed $I_{i}'$. In the last step, $\Pr\left(I_{i}'\left|\mathbf{I},S_{i},\mathbf{\theta}\right.\right)=p_{\Gamma\left(a_{P},b_{S}\right)}\left(S_{i}-I_{j}\right)$ is the cumulative density of the sampling interval distribution, which is the probability of taking proposal path F.

*Acceptance probability*

The acceptance probability (removing the dependency on **θ** after the first line, for readability) is

$$\begin{matrix} \min\left[1,\frac{\Pr\left(\mathbf{S},\mathbf{G},\mathbf{Z}'\left|\mathbf{\theta}\right.\right)\times H\left(\mathbf{Z}\left|\mathbf{Z}',\mathbf{S},\mathbf{\theta}\right.\right)}{\Pr\left(\mathbf{S},\mathbf{G},\mathbf{Z}\left|\mathbf{\theta}\right.\right)\times H\left(\mathbf{Z}'\left|\mathbf{Z},\mathbf{S},\mathbf{\theta}\right.\right)}\right] \\ =\min\left[1,\frac{\Pr\left(\mathbf{G}\left|P'\right.\right)\cdot\Pr\left(P'\left|\mathbf{S},\mathbf{I}',\mathbf{M}'\right.\right)\cdot\Pr\left(\mathbf{S}\left|\mathbf{I}'\right.\right)\cdot\Pr\left(\mathbf{I}',\mathbf{M}'\right)\times\Pr\left(P_{i},P_{j}\left|\mathbf{S},\mathbf{I},\mathbf{M}\right.\right)\cdot0.5\cdot p_{\Gamma\left(a_{P},m_{S}\right)}\left(S_{j}-I_{j}\right)}{\Pr\left(\mathbf{G}\left|P\right.\right)\cdot\Pr\left(P\left|\mathbf{S},\mathbf{I},\mathbf{M}\right.\right)\cdot\Pr\left(\mathbf{S}\left|\mathbf{I}\right.\right)\cdot\Pr\left(\mathbf{I},\mathbf{M}\right)\times\Pr\left(P_{i}',P_{j}'\left|\mathbf{S},\mathbf{I}',\mathbf{M}'\right.\right)\cdot0.5\cdot p_{\Gamma\left(a_{P},m_{S}\right)}\left(S_{i}-I_{j}\right)}\right] \\ =\min\left[1,\frac{\Pr\left(\mathbf{G}\left|P'\right.\right)\cdot\Pr\left(\mathbf{S}\left|\mathbf{I}'\right.\right)\cdot\Pr\left(\mathbf{I}',\mathbf{M}'\right)\cdot p_{\Gamma\left(a_{P},m_{S}\right)}\left(S_{j}-I_{j}\right)}{\Pr\left(\mathbf{G}\left|P\right.\right)\cdot\Pr\left(\mathbf{S}\left|\mathbf{I}\right.\right)\cdot\Pr\left(\mathbf{I},\mathbf{M}\right)\cdot p_{\Gamma\left(a_{P},m_{S}\right)}\left(S_{i}-I_{j}\right)}\right] \end{matrix}$$

*Reversal*

Reversal is possible through proposal path F, with focal host *j*. In Fig 5F, reversal takes place by proposing an infection time for host III after the transmission time to host IV.

**Proposal path G {Tree 2, Q12=YY}**

*Situation*

Host *i* is the index case, and $I_{i}'$ is before its first coalescent node.

*Proposed changes to transmission tree*

1. Topological changes: none
2. Infection time changes:
   1. $I_{i}$ changes to $I_{i}'$

*Proposal steps*

1. bookkeeping: propose $P_{i}'$ by adjusting the infection time

*Proposal distribution*

The proposal distribution for $I_{i}'$ and $P_{i}'$ is

Here, $\Pr\left(P_{i}'\left|...\right.\right)=1$, because it follows automatically from the proposed $I_{i}'$.

*Acceptance probability*

The acceptance probability (removing the dependency on **θ** after the first line, for readability) is

$$\begin{matrix} \min\left[1,\frac{\Pr\left(\mathbf{S},\mathbf{G},\mathbf{Z}'\left|\mathbf{\theta}\right.\right)\times H\left(\mathbf{Z}\left|\mathbf{Z}',\mathbf{S},\mathbf{\theta}\right.\right)}{\Pr\left(\mathbf{S},\mathbf{G},\mathbf{Z}\left|\mathbf{\theta}\right.\right)\times H\left(\mathbf{Z}'\left|\mathbf{Z},\mathbf{S},\mathbf{\theta}\right.\right)}\right] \\ =\min\left[1,\frac{\Pr\left(\mathbf{G}\left|P'\right.\right)\cdot\Pr\left(P'\left|\mathbf{S},\mathbf{I}',\mathbf{M}\right.\right)\cdot\Pr\left(\mathbf{S}\left|\mathbf{I}'\right.\right)\cdot\Pr\left(\mathbf{I}',\mathbf{M}\right)\times d_{\Gamma\left(a_{P},m_{S}\right)}\left(S_{i}-I_{i}\right)}{\Pr\left(\mathbf{G}\left|P\right.\right)\cdot\Pr\left(P\left|\mathbf{S},\mathbf{I},\mathbf{M}\right.\right)\cdot\Pr\left(\mathbf{S}\left|\mathbf{I}\right.\right)\cdot\Pr\left(\mathbf{I},\mathbf{M}\right)\times d_{\Gamma\left(a_{P},m_{S}\right)}\left(S_{i}-I_{i}'\right)}\right] \\ =\min\left[1,\frac{\Pr\left(P_{i}'\left|S_{i},\mathbf{I}',\mathbf{M}\right.\right)\cdot\Pr\left(S_{i}\left|I_{i}'\right.\right)\cdot\Pr\left(\mathbf{I}',\mathbf{M}\right)\cdot d_{\Gamma\left(a_{P},m_{S}\right)}\left(S_{i}-I_{i}\right)}{\Pr\left(P_{i}\left|S_{i},\mathbf{I},\mathbf{M}\right.\right)\cdot\Pr\left(S_{i}\left|I_{i}\right.\right)\cdot\Pr\left(\mathbf{I},\mathbf{M}\right)\cdot d_{\Gamma\left(a_{P},m_{S}\right)}\left(S_{i}-I_{i}'\right)}\right] \end{matrix}$$

Here, $\Pr\left(\mathbf{G}\left|P'\right.\right)=\Pr\left(\mathbf{G}\left|P\right.\right)$, because it does not depend on the infection time of the index case.

*Reverse proposal*

This sampling step can be reversed by proposing through (the same) proposal path G, with the original infection time *I_i_* proposed for the same focal host *i* (similar to path A, Fig 5A).

**Proposal path H {Tree 2, Q12=NY}**

*Situation*

Host *i* is not the index case, and $I_{i}'$ is after the MRCA of the sampling nodes in hosts *i* and $M_{i}$.

*Proposed changes to transmission tree*

1. Topological changes:
   1. transmission nodes move between host *i* and its infector, if $I_{i}$ and $I_{i}'$ are on different branches in the phylogenetic tree *P*
2. Infection time changes:
   1. $I_{i}$ changes to $I_{i}'$

*Proposal steps*

1. bookkeeping: change *h_x_* for all nodes *x* involved: if $I_{i}$ and $I_{i}'$ are on different branches in the phylogenetic tree *P*, coalescent nodes and transmission nodes move from host *i* to host *M_i_* if $I_{i}'>I_{i}$, and vice versa if $I_{i}'<I_{i}$.

*Proposal distribution*

The proposal distribution for $I_{i}'$, $P_{i}'$, and $P_{M_{i}}'$ is

Here, $\Pr\left(P_{i}',P_{M_{i}}'\left|...\right.\right)=1$ and $\Pr\left(\mathbf{M}'\left|...\right.\right)=1$ because these follow automatically from the proposed $I_{i}'$.

*Acceptance probability*

The acceptance probability (removing the dependency on **θ** after the first line, for readability) is

$$\begin{matrix} \min\left[1,\frac{\Pr\left(\mathbf{S},\mathbf{G},\mathbf{Z}'\left|\mathbf{\theta}\right.\right)\times H\left(\mathbf{Z}\left|\mathbf{Z}',\mathbf{S},\mathbf{\theta}\right.\right)}{\Pr\left(\mathbf{S},\mathbf{G},\mathbf{Z}\left|\mathbf{\theta}\right.\right)\times H\left(\mathbf{Z}'\left|\mathbf{Z},\mathbf{S},\mathbf{\theta}\right.\right)}\right] \\ =\min\left[1,\frac{\Pr\left(\mathbf{G}\left|P'\right.\right)\cdot\Pr\left(P'\left|\mathbf{S},\mathbf{I}',\mathbf{M}'\right.\right)\cdot\Pr\left(\mathbf{S}\left|\mathbf{I}'\right.\right)\cdot\Pr\left(\mathbf{I}',\mathbf{M}'\right)\times d_{\Gamma\left(a_{P},m_{S}\right)}\left(S_{i}-I_{i}\right)}{\Pr\left(\mathbf{G}\left|P\right.\right)\cdot\Pr\left(P\left|\mathbf{S},\mathbf{I},\mathbf{M}\right.\right)\cdot\Pr\left(\mathbf{S}\left|\mathbf{I}\right.\right)\cdot\Pr\left(\mathbf{I},\mathbf{M}\right)\times d_{\Gamma\left(a_{P},m_{S}\right)}\left(S_{i}-I_{i}'\right)}\right] \\ =\min\left[1,\frac{\Pr\left(P_{i}',P_{M_{i}}'\left|S_{i},\mathbf{I}',\mathbf{M}'\right.\right)\cdot\Pr\left(S_{i}\left|I_{i}'\right.\right)\cdot\Pr\left(\mathbf{I}',\mathbf{M}'\right)\cdot d_{\Gamma\left(a_{P},m_{S}\right)}\left(S_{i}-I_{i}\right)}{\Pr\left(P_{i},P_{M_{i}}\left|S_{i},\mathbf{I},\mathbf{M}\right.\right)\cdot\Pr\left(S_{i}\left|I_{i}\right.\right)\cdot\Pr\left(\mathbf{I},\mathbf{M}\right)\cdot d_{\Gamma\left(a_{P},m_{S}\right)}\left(S_{i}-I_{i}'\right)}\right] \end{matrix}$$

Here, $\Pr\left(\mathbf{G}\left|P'\right.\right)=\Pr\left(\mathbf{G}\left|P\right.\right)$, because the phylogenetic tree as a whole does not change.

*Reverse proposal*

This sampling step can be reversed by proposing through (the same) proposal path H, with the original infection time *I_i_* proposed for the same focal host *i* (going back in Fig 6A).

**Proposal path I {Tree 2, Q1234=NNYY}**

*Situation*

Host *M_i_* is the index case, $I_{i}'$ is before the MRCA of the sampling nodes in hosts *i* and $M_{i}$, and $I_{M_{i}}'$ is after the MRCA of the sampling nodes *i* and $M_{i}$.

*Proposed changes to transmission tree*

1. Topological changes:
   1. host *i* becomes the index case, and host $M_{i}$ its secondary case
   2. transmission nodes move from host $M_{i}$ to host *i*, consistent with the branch on which $I_{M_{i}}'$ is placed
2. Infection time changes:
   1. $I_{i}$ changes to $I_{M_{i}}$
   2. $I_{M_{i}}$ changes to $I_{M_{i}}'$

*Proposal steps* (after steps 1 and 2 to propose $I_{i}'$ and $I_{M_{i}}'$)

1. discard the proposed $I_{i}'$; instead, propose $I_{i}'=I_{M_{i}}$
2. host *i* becomes the index case: $M_{i}'=0$
3. host *M_i_* gets host *i* as infector: $M_{M_{i}}'=i$
4. bookkeeping: propose other new infectors $\mathbf{M}'$ by changing *h_x_* for all nodes *x* involved: some coalescent nodes and transmission nodes move from host *M_i_* to host *i*.

*Proposal distribution*

The proposal distribution for $I_{i}'$, $I_{M_{i}}'$, $\mathbf{M}'$, $P_{i}'$, and $P_{M_{i}}'$ is

Here, $\Pr\left(P_{i}',P_{M_{i}}'\left|...\right.\right)=1$ and $\Pr\left(\mathbf{M}'\left|...\right.\right)=1$ because these follow automatically from the proposed $I_{i}'$ and $I_{M_{i}}'$. In the last step, $1-p_{\Gamma\left(a_{P},m_{S}\right)}\left(S_{i}-t_{mrca\left(i,M_{i}\right)}\right)$ is 1 minus the cumulative density of the proposal distribution, which is the probability of taking proposal path I (conditional on $I_{M_{i}}'>t_{mrca\left(i,M_{i}\right)}$).

*Acceptance probability*

The acceptance probability (removing the dependency on **θ** after the first line, for readability) is

$$\begin{matrix} \min\left[1,\frac{\Pr\left(\mathbf{S},\mathbf{G},\mathbf{Z}'\left|\mathbf{\theta}\right.\right)\times H\left(\mathbf{Z}\left|\mathbf{Z}',\mathbf{S},\mathbf{\theta}\right.\right)}{\Pr\left(\mathbf{S},\mathbf{G},\mathbf{Z}\left|\mathbf{\theta}\right.\right)\times H\left(\mathbf{Z}'\left|\mathbf{Z},\mathbf{S},\mathbf{\theta}\right.\right)}\right] \\ =\min\left[1,\frac{\Pr\left(\mathbf{G}\left|P'\right.\right)\cdot\Pr\left(P'\left|\mathbf{S},\mathbf{I}',\mathbf{M}'\right.\right)\cdot\Pr\left(\mathbf{S}\left|\mathbf{I}'\right.\right)\cdot\Pr\left(\mathbf{I}',\mathbf{M}'\right)\times\left(1-p_{\Gamma\left(a_{P},m_{S}\right)}\left(S_{M_{i}}-t_{mrca\left(i,M_{i}\right)}\right)\right)\cdot d_{\Gamma\left(a_{P},m_{S}\right)}\left(S_{i}-I_{i}\right)}{\Pr\left(\mathbf{G}\left|P\right.\right)\cdot\Pr\left(P\left|\mathbf{S},\mathbf{I},\mathbf{M}\right.\right)\cdot\Pr\left(\mathbf{S}\left|\mathbf{I}\right.\right)\cdot\Pr\left(\mathbf{I},\mathbf{M}\right)\times\left(1-p_{\Gamma\left(a_{P},m_{S}\right)}\left(S_{i}-t_{mrca\left(i,M_{i}\right)}\right)\right)\cdot d_{\Gamma\left(a_{P},m_{S}\right)}\left(S_{M_{i}}-I_{M_{i}}'\right)}\right] \\ =\min\left[1,\frac{\Pr\left(P_{i}',P_{M_{i}}'\left|S_{i},S_{M_{i}},\mathbf{I}',\mathbf{M}'\right.\right)\cdot\Pr\left(S_{i},S_{M_{i}}\left|I_{i}',I_{M_{i}}'\right.\right)\cdot\Pr\left(\mathbf{I}',\mathbf{M}'\right)\cdot\left(1-p_{\Gamma\left(.\right)}\left(S_{M_{i}}-t_{mrca\left(i,M_{i}\right)}\right)\right)\cdot d_{\Gamma\left(.\right)}\left(S_{i}-I_{i}\right)}{\Pr\left(P_{i},P_{M_{i}}\left|S_{i},S_{M_{i}},\mathbf{I},\mathbf{M}\right.\right)\cdot\Pr\left(S_{i},S_{M_{i}}\left|I_{i},I_{M_{i}}\right.\right)\cdot\Pr\left(\mathbf{I},\mathbf{M}\right)\cdot\left(1-p_{\Gamma\left(.\right)}\left(S_{i}-t_{mrca\left(i,M_{i}\right)}\right)\right)\cdot d_{\Gamma\left(.\right)}\left(S_{M_{i}}-I_{M_{i}}'\right)}\right] \end{matrix}$$

*Reverse proposal*

This sampling step can be reversed by proposing through (the same) proposal path I, with the original index case (now secondary case) as focal host. In Fig 6B, reversal occurs by first proposing any infection time before MRCA_I,II_ for host I, and then proposing for host II its original infection time.

**Proposal path J {Tree 2, Q12345=NNYNY}**

*Situation*

Hosts *i* and *M_i_* are not the index case, $I_{i}'$ is before the MRCA of the sampling nodes in hosts *i* and $M_{i}$ but after the MRCA of the sampling nodes in hosts *i* and $M_{M_{i}}$, and $I_{M_{i}}'$ is after the MRCA of the sampling nodes in hosts *i* and $M_{i}$.

*Proposed changes to transmission tree*

1. Topological changes:
   1. host $M_{M_{i}}$ becomes the infector of host *i*
   2. host *i* becomes the infector of host $M_{i}$
   3. transmission nodes move from hosts $M_{i}$ and $M_{M_{i}}$ to host *i*, consistent with the branches on which $I_{i}'$ and $I_{M_{i}}'$ are placed
2. Infection time changes:
   1. $I_{i}$ changes to $I_{i}'$
   2. $I_{M_{i}}$ changes to $I_{M_{i}}'$

*Proposal steps* (after steps 1 and 2 to propose $I_{i}'$ and $I_{M_{i}}'$)

1. switch role in transmission tree by proposing $M_{i}'=M_{M_{i}}$ and $M_{M_{i}}'=i$
2. bookkeeping: propose other new infectors $\mathbf{M}'$ by changing *h_x_* for all nodes *x* involved: some coalescent nodes and transmission nodes move from hosts $M_{i}$ and $M_{M_{i}}$ to host *i*.

*Proposal distribution*

The proposal distribution for $I_{i}'$, $I_{M_{i}}'$, $P_{i}'$, and $P_{M_{i}}'$ is

Here, $\Pr\left(P_{i}',P_{M_{i}}'\left|...\right.\right)=1$ and $\Pr\left(\mathbf{M}'\left|...\right.\right)=1$ because these follow automatically from the proposed $I_{i}'$ and $I_{M_{i}}'$.

*Acceptance probability*

The acceptance probability (removing the dependency on **θ** after the first line, for readability) is

$$\begin{matrix} \min\left[1,\frac{\Pr\left(\mathbf{S},\mathbf{G},\mathbf{Z}'\left|\mathbf{\theta}\right.\right)\times H\left(\mathbf{Z}\left|\mathbf{Z}',\mathbf{S},\mathbf{\theta}\right.\right)}{\Pr\left(\mathbf{S},\mathbf{G},\mathbf{Z}\left|\mathbf{\theta}\right.\right)\times H\left(\mathbf{Z}'\left|\mathbf{Z},\mathbf{S},\mathbf{\theta}\right.\right)}\right] \\ =\min\left[1,\frac{\Pr\left(\mathbf{G}\left|P'\right.\right)\cdot\Pr\left(P'\left|\mathbf{S},\mathbf{I}',\mathbf{M}'\right.\right)\cdot\Pr\left(\mathbf{S}\left|\mathbf{I}'\right.\right)\cdot\Pr\left(\mathbf{I}',\mathbf{M}'\right)\times d_{\Gamma\left(a_{P},m_{S}\right)}\left(S_{i}-I_{i}\right)\cdot d_{\Gamma\left(a_{P},m_{S}\right)}\left(S_{M_{i}}-I_{M_{i}}\right)}{\Pr\left(\mathbf{G}\left|P\right.\right)\cdot\Pr\left(P\left|\mathbf{S},\mathbf{I},\mathbf{M}\right.\right)\cdot\Pr\left(\mathbf{S}\left|\mathbf{I}\right.\right)\cdot\Pr\left(\mathbf{I},\mathbf{M}\right)\times d_{\Gamma\left(a_{P},m_{S}\right)}\left(S_{i}-I_{i}'\right)\cdot d_{\Gamma\left(a_{P},m_{S}\right)}\left(S_{M_{i}}-I_{M_{i}}'\right)}\right] \\ =\min\left[1,\frac{\Pr\left(P_{i}',P_{M_{i}}'\left|S_{i},S_{M_{i}},\mathbf{I}',\mathbf{M}'\right.\right)\cdot\Pr\left(S_{i},S_{M_{i}}\left|I_{i}',I_{M_{i}}'\right.\right)\cdot\Pr\left(\mathbf{I}',\mathbf{M}'\right)\cdot d_{\Gamma\left(a_{P},m_{S}\right)}\left(S_{i}-I_{i}\right)\cdot d_{\Gamma\left(a_{P},m_{S}\right)}\left(S_{M_{i}}-I_{M_{i}}\right)}{\Pr\left(P_{i},P_{M_{i}}\left|S_{i},S_{M_{i}},\mathbf{I},\mathbf{M}\right.\right)\cdot\Pr\left(S_{i},S_{M_{i}}\left|I_{i},I_{M_{i}}\right.\right)\cdot\Pr\left(\mathbf{I},\mathbf{M}\right)\cdot d_{\Gamma\left(a_{P},m_{S}\right)}\left(S_{i}-I_{i}'\right)\cdot d_{\Gamma\left(a_{P},m_{S}\right)}\left(S_{M_{i}}-I_{M_{i}}'\right)}\right] \end{matrix}$$

*Reverse proposal*

This sampling step can be reversed by proposing through (the same) proposal path J, with the original infector (now secondary case) as focal host. In Fig 6C, reversal occurs by first proposing the original infection time for host II (which is between the two MRCAs), and then proposing the original infection time for host III.

**Proposal path K {Tree 3}**

*Situation*

Any.

*Proposed changes to transmission tree*

None.

*Proposal steps*

1. discard the sampled infection time $I_{i}'$
2. propose new tree $P_{i}'$ by simulating only new $v_{x\left|x\in P_{i}\right.}$ (a new topology, not coalescent times)

*Proposal distribution*

The proposal distribution for $P_{i}'$ is

$$H\left(P_{i}'\left|\mathbf{I},\mathbf{M},P,\mathbf{S},\mathbf{\theta}\right.\right)=\Pr\left(P_{i}'\left|\mathbf{I},\mathbf{M},S_{i},\mathbf{\theta}\right.\right)$$

*Acceptance probability*

The acceptance probability (removing the dependency on **θ** after the first line, for readability) is

$$\begin{matrix} \min\left[1,\frac{\Pr\left(\mathbf{S},\mathbf{G},\mathbf{Z}'\left|\mathbf{\theta}\right.\right)\times H\left(\mathbf{Z}\left|\mathbf{Z}',\mathbf{S},\mathbf{\theta}\right.\right)}{\Pr\left(\mathbf{S},\mathbf{G},\mathbf{Z}\left|\mathbf{\theta}\right.\right)\times H\left(\mathbf{Z}'\left|\mathbf{Z},\mathbf{S},\mathbf{\theta}\right.\right)}\right] \\ =\min\left[1,\frac{\Pr\left(\mathbf{G}\left|P'\right.\right)\cdot\Pr\left(P'\left|\mathbf{S},\mathbf{I},\mathbf{M}\right.\right)\cdot\Pr\left(\mathbf{S}\left|\mathbf{I}\right.\right)\cdot\Pr\left(\mathbf{I},\mathbf{M}\right)\times\Pr\left(P_{i}\left|\mathbf{S},\mathbf{I},\mathbf{M}\right.\right)}{\Pr\left(\mathbf{G}\left|P\right.\right)\cdot\Pr\left(P\left|\mathbf{S},\mathbf{I},\mathbf{M}\right.\right)\cdot\Pr\left(\mathbf{S}\left|\mathbf{I}\right.\right)\cdot\Pr\left(\mathbf{I},\mathbf{M}\right)\times\Pr\left(P_{i}'\left|\mathbf{S},\mathbf{I},\mathbf{M}\right.\right)}\right] \\ =\min\left[1,\frac{\Pr\left(\mathbf{G}\left|P'\right.\right)}{\Pr\left(\mathbf{G}\left|P\right.\right)}\right] \end{matrix}$$

*Reversal*

Reversal is possible through the same proposal path K, by resampling the original $P_{i}$.

Irreducibility of the MCMC chain

Here we argue heuristically that the MCMC chain is irreducible, i.e. any configuration of the transmission tree and phylogenetic tree consistent with the sampling times can be reached from any (current) configuration:

- for every host *i*, it is possible to reach any infection time *I_i_* (prior to its sampling time *S_i_*), without changing the other hosts’ infection times:
  - if host *i* does currently not have secondary cases:
    - sample any infection time with host *i* as focal host, followed by proposal path D or E
  - if host *i* does currently have secondary cases, but is not the index case:
    - first, lose all secondary cases of host *i* by taking these secondary cases as focal hosts, sampling the infection times they already have, thus following proposal path E, and proposing alternative infectors
    - then, sample any infection time with host *i* as focal host, followed by proposal path D or E
  - if host *i* is currently the index case:
    - first, lose the index case status by a single proposal path B with host *i* as focal host
    - then, lose all secondary cases of host *i* by taking these secondary cases as focal hosts, sampling the infection times they already have, thus following proposal path E, and proposing alternative infectors
    - then, sample any infection time with host *i* as focal host, followed by proposal path D or E
- for every set of infection times **I**, all transmission trees consistent with those times can be reached:
  - with host *i* as focal host, sample the infection time it already has, and follow proposal path A (if host *i* is index case) or proposal path E (otherwise), and propose any host infected before *I_i_* as infector.
- for every transmission tree, all phylogenetic trees consistent with that tree can be reached:
  - with host *i* as focal host, sample the infection time it already has, follow proposal path A (if host *i* is index case) or proposal path E (otherwise), sample the infector it already has, and simulate the phylogenetic minitree in host *i* (and its infector).

**References**

1. Felsenstein J. Evolutionary trees from DNA sequences: a maximum likelihood approach. J Mol Evol. 1981;17(6):368-76. PubMed PMID: 7288891.

2. Roberts GO, Gelman A, Gilks WR. Weak convergence and optimal scaling of random walk metropolis algorithms. Ann Appl Prob. 1997;7(1):110-20.
